# Synthesis, Characterization, DFT Study and Antifungal Activities of Some Novel 2-(Phenyldiazenyl)phenol Based Azo Dyes

**DOI:** 10.3390/ma15228162

**Published:** 2022-11-17

**Authors:** Maria Marinescu, Claudia Valentina Popa, Maria Antonia Tănase, Andreia Cristina Soare, Cristina Tablet, Daniela Bala, Ludmila Otilia Cinteza, Lia Mara Diţu, Ioana Catalina Gifu, Cristian Petcu

**Affiliations:** 1 Department of Organic Chemistry, Biochemistry and Catalysis, Faculty of Chemistry, University of Bucharest, 90 Soseaua Panduri, 050663 Bucharest, Romania; 2 Laboratory of Pharmaco-Toxicology, National Institute for Medical Military Research Development “Cantacuzino”, 103 Splaiul Independentei, 050096 Bucharest, Romania; 3 Physical Chemistry Department, Faculty of Chemistry, University of Bucharest, 4-12 Blv. Regina Elisabeta, 030018 Bucharest, Romania; 4 Faculty of Pharmacy, Titu Maiorescu University, 16 Gh. Sincai Blvd, 040317 Bucharest, Romania; 5 Microbiology Department, Faculty of Biology, University of Bucharest, 3 Intrarea Portocalelor, 60101 Bucharest, Romania; 6 Polymer Department, National Institute for Research and Development in Chemistry and Petrochemistry-ICECHIM, 202 Spl. Independentei, 060021 Bucharest, Romania

**Keywords:** azo dyes, antifungal, fluorescence, DFT

## Abstract

In recent decades, there has been an increased interest in azo compounds with special optical and biological properties. In this work, we report the preparation of novel azo-compounds with two and three –N=N- double bonds, using the classical method of synthesis, diazotization and coupling. The compounds were characterized by ^1^H-NMR, ^13^C-NMR, FTIR, UV-VIS and fluorescence spectra. DFT calculations were employed for determining the optical parameters, polarizability α, the total static dipole moment μ_tot_, the quadrupole moment Q and the mean first polarizability β_tot_. All azo derivatives show strong fluorescence emission in solutions. The antioxidant and antifungal activities were determined and the influence of the number of azo bonds was discussed. The synthesized compounds exhibit remarkable efficiency in the growth reduction of standard and clinical isolated *Candida* strains, suggesting future applications as novel antifungal.

## 1. Introduction

Compounds that contain azo groups linked to methine or aromatic sp^2^-hybridized C-atoms are called azo dyes [1]. The presence of the functional group (−N=N−) that bonds two alkyl or aryl radicals, symmetrical or asymmetric, is characteristic of azo dyes. Thus, azobenzene, an organic molecule made up of two phenyl rings joined by a double bond, is the simplest compound in the class of azo compounds [2]. Azo dyes are a well-known class of compounds, due to their relatively simple accessibility and their various uses [3]. Azo dyes represent the largest total category (60–70%) in the synthetic dyes industry, due to the advantages presented, such as high stability, intensity of the color, cost-effectiveness, versatility, and simplicity of utilization [4].

The researchers explored simple approaches for the synthesis of mono-azo dyes, in two stages (i) diazotization of the aromatic (or heteroaromatic) amine under strongly acidic conditions, with the formation of the diazonium salt; and *(*ii*)* coupling of the diazonium salt carried out at low temperature in the presence of nucleophilic coupling components [5]. The literature reports, in addition to the classic mono-azo dyes [1], dyes with more complex structures, containing two azo groups [6], dyes that possess three azo groups (tris-azo) [7], four azo groups (tetrakis-azo), or in much rarer cases, molecules where there are more than four azo groups (polyazo).

Among the properties of the azo dyes, the most important are optical, on which their application are based as nonlinear optical (NLO) materials [8], laser materials [9], ink-jet inks [10], sensitizers in solar cells [11], optical materials with triggered birefringence [12], photostable disperse dyes for textile fabric [13], cosmetics dyes [14] and food additives [15].

In particular, NLO properties are highly demanded in present developments in the optical material industry. Literature has shown that a couple:donor–π−acceptor (or “push–pull” system) connected to a system which contributes to the delocalization of the π-electrons is the key factor in designing such NLO compounds. Molecular hyperpolarizability is the most important characteristic of such push–pull molecules. Large hyperpolarizabilities arise from a combination of strong electron donor groups (e.g., –NR_2_, –OR), and strong electron withdrawing groups (e.g., –NO_2_, –CN), positioned at opposite ends of a conjugated molecule [16].

Azo compounds usually contain a highly delocalized electron system that takes up both benzene rings and the two nitrogen atoms that bind the rings. The delocalization can also extend to groups attached to benzene rings. Therefore, azo dyes are important chromophores with nonlinear optical (NLO) applications [8].

NLO responses of different azo-dyes were reported in the last decade [17]. Our previous studies referred to the tautomerism of pyrazalone azo-dyes, and their NLO properties of the two conformers, resulting from DFT calculations of several parameters, such as molecular polarizability (α), first order hyperpolarizabilities (β_tot_), HOMO-LUMO gap, dipole (μ_tot_) and quadrupole moments (Q) [18], where HOMO is the highest occupied molecular orbital and LUMO is the lowest unoccupied molecular orbital.

In recent years, there has been a growing interest in the synthesis of new azo dyes, due to the other properties they possess, including medicinal properties, such as antimicrobial, antiviral, anti-inflammatory, anticancer, antioxidant [19,20], and for other applications such as liquid crystals [21] or as biomedical carriers. Supramolecular azo dye was also reported to form complexes with DNA and serum proteins, thus the binding properties significantly influence the biological [22]. Some metal-azo dye complexes (with Fe, Co, Zn and Ru) were recently synthesized and tested as potential drugs in Alzheimer’s disease treatment, due to their properties as cholinesterase inhibitors [23].

The aim of the present work was to synthesize novel azo derivatives with extended structure (bis and tris azo), in order to obtain superior optical and biological properties, using a simple, cost-effective synthesis route. The new chromophore structures are designed with simple functional moieties, in order to accommodate convenient synthesis, as push–pull architectures to produce high polarizabilities, together with antifungal activity.

## 2. Materials and Methods

### 2.1. Materials

All the reagents and solvents (Fluka, Buchs, Switzerland) used in the synthesis of the azo derivatives were employed as received.

Dimethyl-sulfoxide DMSO, spectroscopic grade (Merck Group, Darmstadt, Germany) and bi-distilled water were used as solvent for the preparation of the solutions. Reagents for antioxidant activity were 2.2-Diphenyl-1-picrylhydrazyl radical (DPPH), gallic acid, caffeic acid, curcumin Sigma-Aldrich (Merck Group, Darmstadt, Germany), and ethanol (96%, Chimreactiv, Bucharest, Romania). Bovine serum albumin (BSA), (powder Sigma-Aldrich reagent ≥ 98%, purchased from Merck Group, Darmstadt, Germany), was used as model serum protein in binding studies.

### 2.2. Characterization of Azo Derivatives

NMR spectra were recorded on Bruker Avance III 500 (^1^H: 500 MHz, ^13^C: 125 MHz) and Bruker Avance III HD 600 MHz (^1^H: 600 MHz, ^13^C: 150 MHz) NMR instruments (Bruker Biospin, Ettlingen, Germany). Melting points were determined in open capillary tubes using a STUART SMP3 electric melting point apparatus (Bibby Sterilin Ltd., Stone, UK) and are uncorrected. Elemental analysis was performed with a multi EA 4000 (Analytik Jena, Jena, Germany) device.

Absorption spectra of all azo derivatives were recorded using a Jasco V-660 spectrometer (Jasco Corporation, Tokyo, Japan) in the 200–800 nm range, at room temperature, in a 1 cm quartz cuvette.

Fluorescence emission of the azo derivatives was measured on powder, using a OceanInsight XDH spectrometer (OceanInsight, Orlando, FL, USA) modular device coupled via optical fiber to a LED source emitting at 365 nm on reflection mode, and in solution using a Jasco V850 spectro-fluorimeter (Jasco Corporation, Tokyo, Japan). FTIR spectra were collected using a Tensor 37 Bruker equipment (Bruker Corporation, Billerica, MA, USA) instrument within the spectral range 400–4000 cm^−1^, with 32 scans at a resolution of 4 cm^−1^. Sample pellets were prepared by adding azo dye powder to KBr powder.

### 2.3. DFT Calculations

Optimized geometries, NLO activity and photophysical properties were investigated by DFT calculations performed with Gaussian09 [24] using the CAM-B3LYP [25] functional. For the calculation of hyperpolarizability, dipole moments and polarizabilities, we used the 6-311++G** basis set, while for the simulation of the absorption spectra the 6-31+G** basis [26] was used. Molecular geometries of the ground state were optimized in vacuum and DMSO (PCM model [27]) according to the desired properties. To ensure that they correspond to a minimum point on the potential energy surfaces, the vibrational frequencies were calculated and it was confirmed that there are no frequencies with imaginary values. The values of the first hyperpolarizability were calculated using the polar keyword and data processing was performed with the Multiwfn program [28]. TD-DFT calculations were performed and absorption spectra were simulated using Gabedit software [29]. To clarify the fluorescence spectra in DMSO, we also optimized the geometries of the first singlet state.

### 2.4. Electrochemical Determinations

Electrochemical experiments were performed using a Autolab PGStat 12 potentiostat-galvanostat system (Echo Chemie BV, Utrecht, The Netherlands) controlled by General Purpose Electrochemical System (GPES) electrochemical interface for Windows. Three electrodes in one-compartment cell (10 mL) were used in all experiments: glassy carbon electrode (Metrohm, 3 mm in diameter) as working electrode, Pt wire as counter electrode, respectively, and Ag/AgCl as reference electrode. The glassy carbon electrode surface was polished with alumina slurry on a polishing pad, washed with distilled water and sonicated for 1 min.

The reagents dimethyl-sulfoxide DMSO, ACS reagent was purchased from Sigma-Aldrich (Riedel-de Haen, Frankfurt am Main, Germany), and tetrabutylammonium tetrafluoroborate Bu_4_NBF_4_, for electrochemical analysis was purchased from Sigma-Aldrich (Merck Group, Poole, UK), used as purchased.

Two working solutions of 10 mL each were prepared and studied: one obtained by dissolving the electroactive compounds Mono_A, Di_A or Tris_A (1 mM) in dimethyl-sulfoxide with 0.1 M Bu_4_NBF_4_ as supporting electrolyte. Cyclic and differential pulse voltammetry (CV and DPV) measurements were performed.

For all compounds, cyclic voltammetry experiments were carried out in the potential ranges from –2.8 V to +1.5 V. To investigate the influence of scan rate, several values were used in the range between 0.1 to 1 V s^–1^. DPV curves were recorded on the same potential domains using different parameters: step potential (SP) 50 mV and modulation amplitude (MA) 25 mV.

The solution containing the electroactive species (Mono_A, Di_A or Tris_A) and the supporting electrolyte was purged with Ar, and low-pressure inert gas atmosphere was maintained above the solution during the electrochemical experiments.

### 2.5. Antioxidant Activity

For the determination of DPPH. scaverging activity a DPPH solution 0.010 g in 96% ethanol was used, obtained by dissolving 0.010 g DPPH in 100 mL alcoholic solution. Solutions in ethanol 96% of caffeic acid, gallic acid 1 mg/mL, and curcumin, prepared by dissolving 0.010 g standard for 10 mL of each alcoholic solution, are used as references. The solutions for plotting the calibration curve have concentrations between 0.0293 and 30.0 μg/mL (calibration curve is shown in Supplementary Materials File).

DPPH free radical scavenging activity was carried out according to a procedure addapted from the literature. Briefly, 500 µL standard/sample were mixed with 500 µL DPPH solution (1 mg/mL in ethanol 96%) and the mixture kept in the dark for 30 min. Absorbance measurements were carried out at the wavelength of 536 nm. All samples were worked in triplicate.

Calibration curves inhibition percent (% I) vs. standard concentration (μg/mL) were drawn. Antioxidant activity of samples was expressed as μg standard equivalents/mL sample, and the samples had a concentration of 1 mg compound/mL ethanolic solution.

The antioxidant activity, expressed as percentages of *DPPH* inhibitory activity, was calculated with Equation (1):

(1) $$\%DPPH=\frac{A_{C}-A_{S}}{A_{C}\times 100}$$

where, *A_C_* = *DPPH* absorbance and *A_S_* = sample absorbance.

Data processing was performed in Microsoft Office EXCEL 97-2003 (Microsoft Corporation, Redmond, WA, USA).

### 2.6. BSA Interaction

Interaction between serum protein and the synthesized azo derivatives was investigated based on the fluorescence quenching in the bovine serum albumin (BSA) solution, as model protein, according to a procedure recommended in the literature [30]. A volume of 2 mL of BSA solution with concentration 2 μM in phosphate buffer, pH 7.4, was placed in the 1.0 cm quartz cell and titrated successively with solution of azo compounds with concentration 1 × 10^−4^ M. The fluorescence spectra were obtained with excitation wavelength set at 285 nm and recorded in the region 300 to 500 nm. The measurements were performed at 25 °C, using a Jasco 8200 fluorimeter (Jasco Corporation, Tokyo, Japan).

### 2.7. Drug Likeness and Pharmacokinetics Prediction

The pharmacokinetics parameters and drug likeness characteristics for the novel azo derivatives were computed using SwissADME online tools [31].

### 2.8. Antifungal Activity

The antimicrobial assay was performed using standard (ATCC) and clinical (CL) yeast strains from the University of Bucharest, Microbiology Department collection, as follows: *Candida albicans* ATCC 10231, *Candida albicans* CL 7626, *Candida pasapsilosis* CL 5348, *Candida tropicalis* CL 3301, *Candida glabrata* CL 5328, *Candida krusei* CL 5343.

The qualitative screening of the anti-microbial properties was performed by an adapted spot diffusion method [32]. The yeast suspensions of 1.5 × 10^8^ CFU/mL (corresponding with 1 McFarland standard density) obtained from 48 h yeast cultures were used in the experiments. Petri dishes with Sabouraud media were seeded with microbial inoculums and an amount of 10 µL solution of each sample Mono-azo, Bis-azo, Tris-azo (1 mg/mL), was spotted, including fluconazole (1 mg/mL) as control. The plates were left at room temperature to ensure the equal diffusion of the compound in the medium and then incubated at 37 °C for 24–48 h. Sensitivity was evaluated by measuring the diameters of the inhibition zones that appeared around the spot and expressed in mm.

The quantitative assay was performed in Sabouraud broth, using the binary serial microdilution technique on 96-well microtiter plates, according to Performance Standard for Antimicrobial Susceptibility Testing [33], in order to establish the MIC (minimum inhibitory concentration) values of the tested compounds. The sterile broth was added to sterile 96 well plates and binary dilutions of each tested solution were performed at a final volume of 150 μL, followed by the addition of 15 μL microbial suspension adjusted to an optical density of 0.5 McFarland (1.5 × 10^8^ CFU/mL) in each well. The MIC values were established by visual analysis and spectrophotometric measurement (absorbance reading at 600 nm). Each experiment was performed in triplicate and repeated on at least three separate occasions.

For the quantitative assessment of the inert substratum adherence, in order to determinate the minimal concentration for biofilm eradication values (MBEC), 96-multi well plastic plates containing binary dilutions of the tested compounds, at a final volume of 150 µL broth media, were inoculated with 15 µL microbial suspensions of 10^7^ CFU/mL and incubated for 48 h at 37 °C. After incubation, the wells were discarded, washed three times in PBS and the bacterial cells adhering to the plastic walls were stained by 1% violet crystal solution for 15 min. The colored adherent cells were thereafter fixed by cold methanol for 5 min and re-suspended in 33% acetic acid solution. The absorbance at 490 nm of the blue suspension was measured using BIOTEK SYNERGY-HTX ELISA multi-mode reader (BioTek Instruments, Winooski, VT, USA), the obtained values being proportional to the number of the adhered microbial cells.

## 3. Results

### 3.1. Synthesis of Azo-Compounds

**Figure 1:**
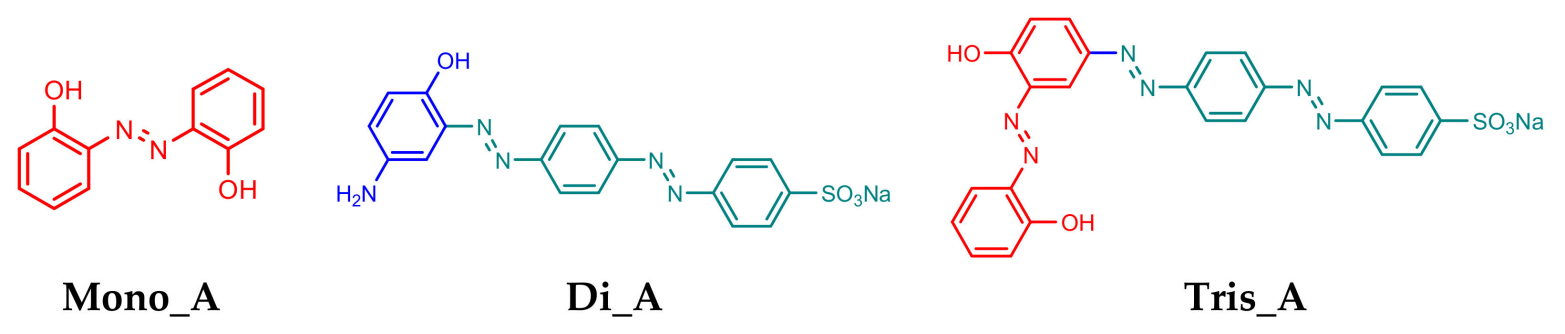
Structures of synthesized azo compounds Mono_A, Di_A and Tris_A.

In this paper three azo-compounds were synthesized using classical diazotization and coupling reactions: a mono-azo derivative (Mono_A), a di-azo-derivative (Di_A) and a tri-azo-derivative (Tris_A) (Figure 1) and their oxidative-reducing, optical and antifungal properties were studied, as well as the characteristics of compounds, by functional density theory (DFT).

**Scheme 1:**
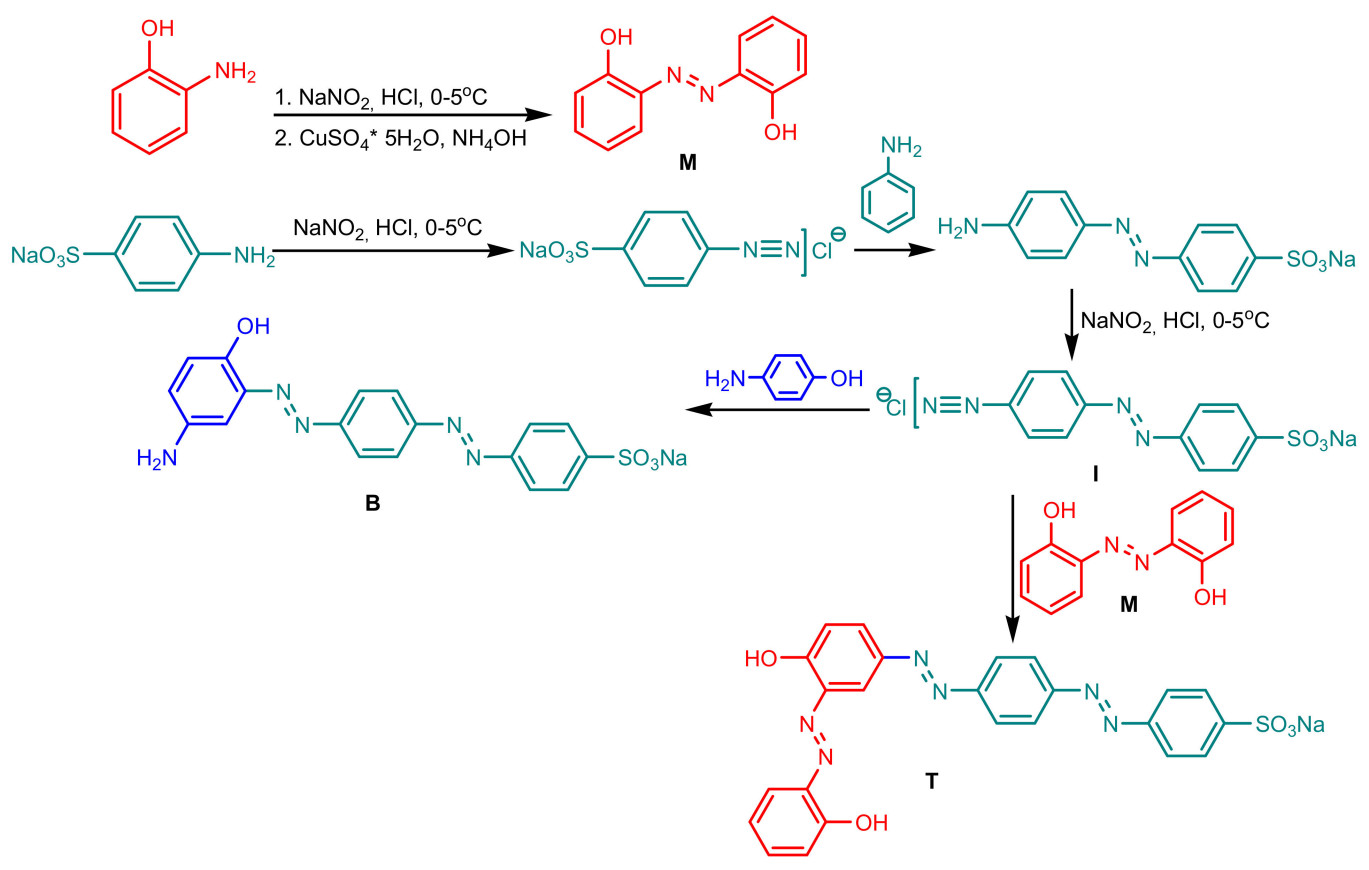
Synthesis of the azo-dyes Mono_A, Di_A and Tris_A.

The synthesis of azo-dyes was performed according to Scheme 1—2,2′-Dihydroxyazobenzene (Mono_A) was synthesized from 2-aminophenol by diazotization and coupling in the presence of the copper catalyst. The diazotization of 4-((4-aminophenyl)diazenyl)benzenesulfonate gave the intermediate diazonium salt (I), from which the diazo compound Di_A was obtained by coupling with 4-aminophenol and triazo compound Tris_A, by coupling with 2.2′-dihydroxyazobenzene. The structures of the compounds were confirmed by ^1^H and ^13^C NMR spectra, absorption spectra and elemental analysis.

#### 3.1.1. Synthesis of 2,2′-Dihydroxyazobenzene (Mono_A)

A mixture of 1.1 mmol 2-aminophenol in 37% HCl (5 mL) was stirred in an ice-bath, and then the solution of 1.1 mmol sodium nitrite in water (5 mL) was added slowly (the temperature was not allowed to rise above 5 °C) for the formation of diazonium salt, which was allowed to react with the catalyst solution with constant stirring. The catalyst solution was prepared as follows: copper (II) sulfate pentahydrate (2.85 g) was dissolved in 10 mL hot water. The solution was cooled and treated with concentrated ammonium hydroxide until the formation of the soluble ammonia complex (I) was complete. After that, 0.7 g hydroxylamine hydrochloride in 2 mL water was added to complex (I) to be reduced to a colorless solution of copper (I). The reaction mixture was stirred at 0–5 °C for 60 min. and then under an ambient temperature for 30 min. The reaction progress was monitored by thin-layer chromatography (TLC) using a mixture of EtOAc and n-hexane (1:1; v/v) as a solvent. Following the disappearance of the starting materials, the reaction mixture was filtrated and washed with water. The crude solid was recrystallized from benzene to give a 78% yield of yellow-orange needles that melted at 172–172.5 °C (Lit. 172 °C) [19]. ^1^H-NMR, (600,12 MHz, DMSO, δ(ppm), J(Hz)): 11.71 ppm, (s, 2-^1^H, H-1, H-1′); 7.85 ppm, (dd, 2-^1^H, 6.5 Hz, 8 Hz, H-3, H-3′); 7.40 ppm (t, 2-^1^H, 8.5 Hz, H-5, H-5′); 7.07 ppm (d, 2-^1^H, 8.2 Hz, H-4, H-4′); 7.02 ppm (t, 2-^1^H, 7.8 Hz, H-12). ^13^C-NMR (150,9 MHz, DMSO, δ(ppm)): 153.9 ppm, C-2, C-2′; 137.6 ppm, C-7, C-7′; 133.2 ppm C-5, C-5′; 123.2 ppm C-3, C-3′; 119.8 ppm, C-4, C-4′; 118 ppm, C-6, C-6′. Elemental analysis (%) found for C_12_H_10_N_2_O_2_: C, 67.24; H, 4.68; N, 13.11; O, 13.05. calcd.: C, 67.28; H, 4.71; N, 13.08; O, 14.94.

#### 3.1.2. Synthesis of Sodium 4-((E)-(4-((E)-(5-Amino-2-hydroxyphenyl)diazenyl)phenyl)diazenyl)benzene Sulfonate (Di_A)

A mixture of 1.1 mmol sodium 4-((4-aminophenyl)diazenyl)benzenesulfonate in 37% HCl (5 mL) was stirred in an ice-bath, and then the solution of 1.1 mmol sodium nitrite in water (5 mL) was added slowly (the temperature was not allowed to rise above 5 °C) for the formation of diazonium salt (**I**). The diazonium salt was added over a 1 mmol solution of 4-aminophenol dissolved in a 10% NaOH solution. The reaction progress was monitored by thin-layer chromatography (TLC) using a mixture of EtOAc and n-hexane (1:1; v/v) as a solvent. Following the disappearance of the starting materials, the reaction mixture was filtrated and washed with water. The crude solid was recrystallized from toluene to give a 93% yield of red-brown needles that melted at 280–282 °C. ^1^H-NMR, (500 MHz, DMSO, δ(ppm), J(Hz)): 8.54 ppm (s, OHaromatic); 7.91 ppm (d, 2-^1^H, 10 Hz); 7.71 ppm (m, 4-^1^H); 7.61 ppm (d, 2-^1^H, 10 Hz); 7.2 ppm (s, ^1^H); 7.67 ppm (d, 2-^1^H, 10 Hz); 6,13 ppm (s, NH_2_ aromatic). ^13^C-NMR (150,9 MHz, DMSO, δ(ppm)): 157.30; 156.08; 153.35; 142.68; 141.48; 128.45; 126.68; 126.45; 125.20; 122.05; 121.01; 113.35 ppm. Elemental analysis (%) found for C_18_H_14_N_5_NaO_4_S: C, 51.52; H, 3.34; N, 16.73; Na, 5.47; O, 5.27; S, 7.67. calcd.: C, 51.55; H, 3.36; N, 16.70; Na, 5.48; O, 5.26; S, 7.65.

#### 3.1.3. Synthesis of Sodium 4-((4-((4-Hydroxy-3-(2-hydroxyphenyl)diazenyl)phenyl)diazenyl)phenyl)diazenyl)Benzene Sulfonate (Tris_A)

A mixture of 1.1 mmol sodium 4-((4-aminophenyl)diazenyl)benzenesulfonate in 37% HCl (5 mL) was stirred in an ice-bath, and then the solution of 1.1 mmol sodium nitrite in water (5 mL) was added slowly (the temperature was not allowed to rise above 5 °C) for the formation of diazonium salt (**I**). The diazonium salt was added over a 1 mmol solution of 2,2′-(diazene-1,2-diyl)diphenol dissolved in a 10% NaOH solution. The reaction progress was monitored by thin-layer chromatography (TLC) using a mixture of EtOAc and n-hexane (1:1; v/v) as a solvent. Following the disappearance of the starting materials, the reaction mixture was filtrated and washed with water. The crude solid was recrystallized from toluene to give a 91% yield of dark green needles with melting point above of 300 °C. ^1^H-NMR, (500 MHz, DMSO, δ(ppm), J(Hz)): 8.54 ppm (s, OHaromatic); 7.91 ppm (d, 2-^1^H, 10 Hz); 7.71 ppm (m, 4-^1^H); 7.61 ppm (d, 2-^1^H, 10 Hz); 7.2 ppm (s, ^1^H); 7.67 ppm (d, 2-^1^H, 10 Hz); 6,13 ppm (s, NH_2_ aromatic). ^13^C-NMR (150,9 MHz, DMSO, δ(ppm)): 157.30; 156.08; 153.35; 142.68; 141.48; 128.45; 126.68; 126.45; 125.20; 122.05; 121.01; 113.35 ppm. Elemental analysis (%) found for C_24_H_17_N_6_NaO_5_S: C, 54.94; H, 3.25; N, 16.04; Na, 4.37; O, 15.27; S, 6.12. calcd.: C, 54.96; H, 3.27; N, 16.02; Na, 4.38; O, 15.25; S, 6.11.

The structures of all the compounds were confirmed by ^1^H, and ^13^C nuclear magnetic resonance (NMR) and elemental analysis, as described above. In the ^1^H NMR spectrum of Mono_A, the aromatic hydrogens of the benzene ring in the structure were observed as peaks between δ = 7.01 ppm÷7.85 ppm and the hydrogen of the OH groups at δ = 11.70 ppm. For Di_A, the amino hydrogens at δ = 6.13 ppm are the most shielded, followed by the doublet for the two protons between amino and hydroxy group, at δ = 6.67 ppm, and the singlet at δ = 7.20 ppm for aromatic hydrogen neighbor with NH_2_ group. The most de-shielded aromatic hydrogens, between δ = 7.54 ppm–7.92 ppm correspond to the aromatic hydrogens from the unsubstituted central nucleus (due to the presence of the two azo groups in the para position) and from the benzene-sulfonated moiety. The chemical shift from δ = 8.54 ppm is attributed to the −OH group, which forms hydrogen bonds with the neighboring azo group. In the ^1^H NMR spectrum of compound Tris_A, we can see the chemical shift from δ = 11.77 ppm for the phenolic hydrogens (−OH) and the peaks between 6.67–8.51 ppm for the hydrogens from the aromatic nuclei.

### 3.2. DFT Study

**Figure 2:**
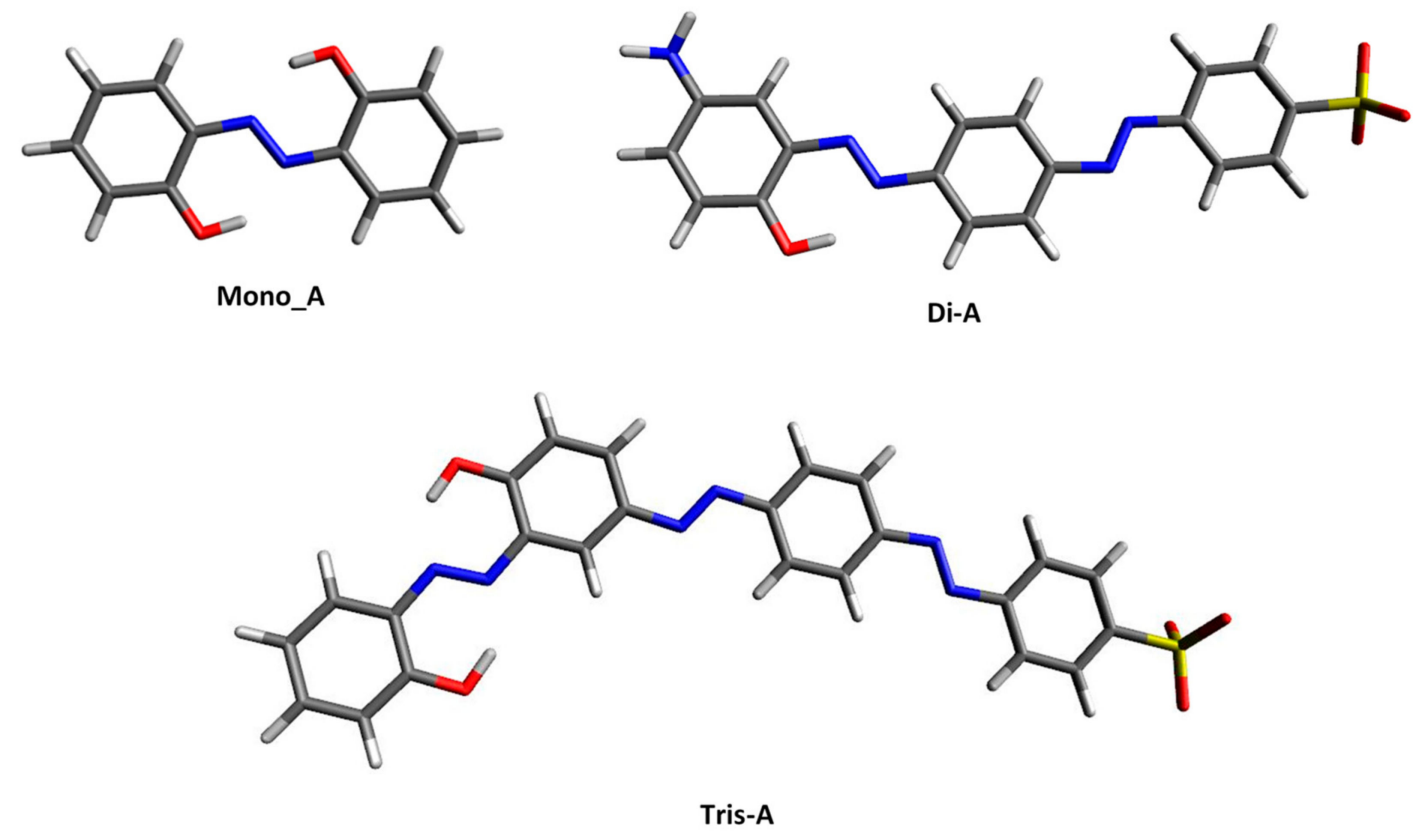
Ground state optimized structures in vacuo for the studied azo-derivates.

The most stable ground-state structures of the vacuum optimized azo derivatives are shown in Figure 2. Some of their important structural features, such as interatomic distance, valence angle and torsion angle, are listed in the Table S1 in Supplementary Materials File. As can be seen, the studied molecules exist in an all-trans planar form and adopt a conformation that allows both the azo nitrogen and the −OH group to exist in a six-membered ring-like structure.

**Figure 3:**
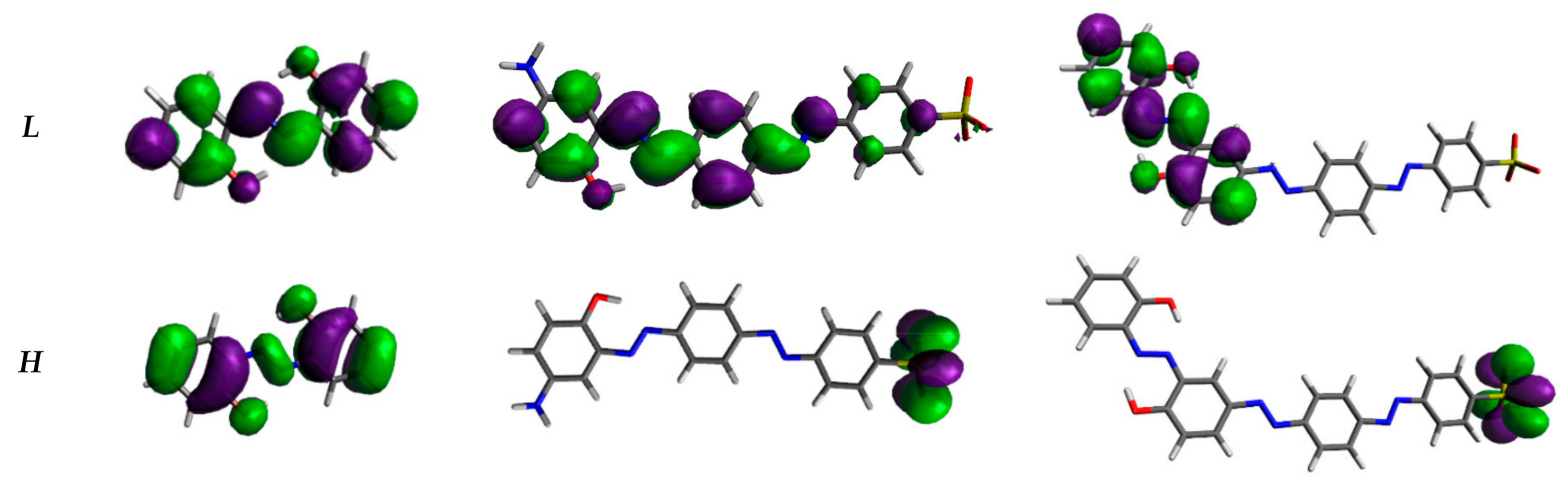
Shapes of the frontier orbitals of our azo-derivatives calculated at CAM-B3LYP/6-311++G** level *in vacuo*. H stands for HOMO and L for LUMO. Iso-density value: 0.02 e/Bohr^3^.

The analysis of molecular frontier orbitals (Figure 3) provides first indications of the hyperpolarizability of compounds. Bis-azo and tris-azo have a slight energy difference between homo (H) and lumo (L) (ΔE_H-L_) (Table 1), facilitating effective charge transfer: ***H*** is only on the sulfonic group, while ***L*** is localized on the remaining fragments. Consequently, these azo derivatives show high hyperpolarizability, which increases with ΔE_H-L_. Similar behaviour has recently been reported in the literature for various azo-derivatives [34].

Starting from the optimized structures in vacuum, the first static hyperpolarizability (β) and three related properties were computed: the mean isotropic polarizability (α), the polarizability anisotropy (Δα), and the dipole moment (μ). Table 1 gives their values. It can be seen that, with increasing values for α, Δα and μ, β also increases. While Di-azo and Tris-azo have very large β values, Mono-azo has very low hyperpolarizability, consistent with a larger *ΔE_H-L_* and lack of charge transfer. Thus, with the incorporation of two or three double bonds –N=N- in a suitably designed chemical structure, significant increase in the NLO properties could be expected for the Di_A and Tris_A novel compounds.

In the ground state, azo derivatives are more stable in the E configuration. Once heated or exposed to the right wavelength, they can be converted into a Z-shape. However, when the hydroxyl is in ortho to the azo group, tautomerization is favored over isomerization [35]. In the excited state, this tendency can be even more pronounced, which can lead to ESIPT (Excited State Intramolecular Proton Transfer) [36], *vide infra*.

**Figure 4:**
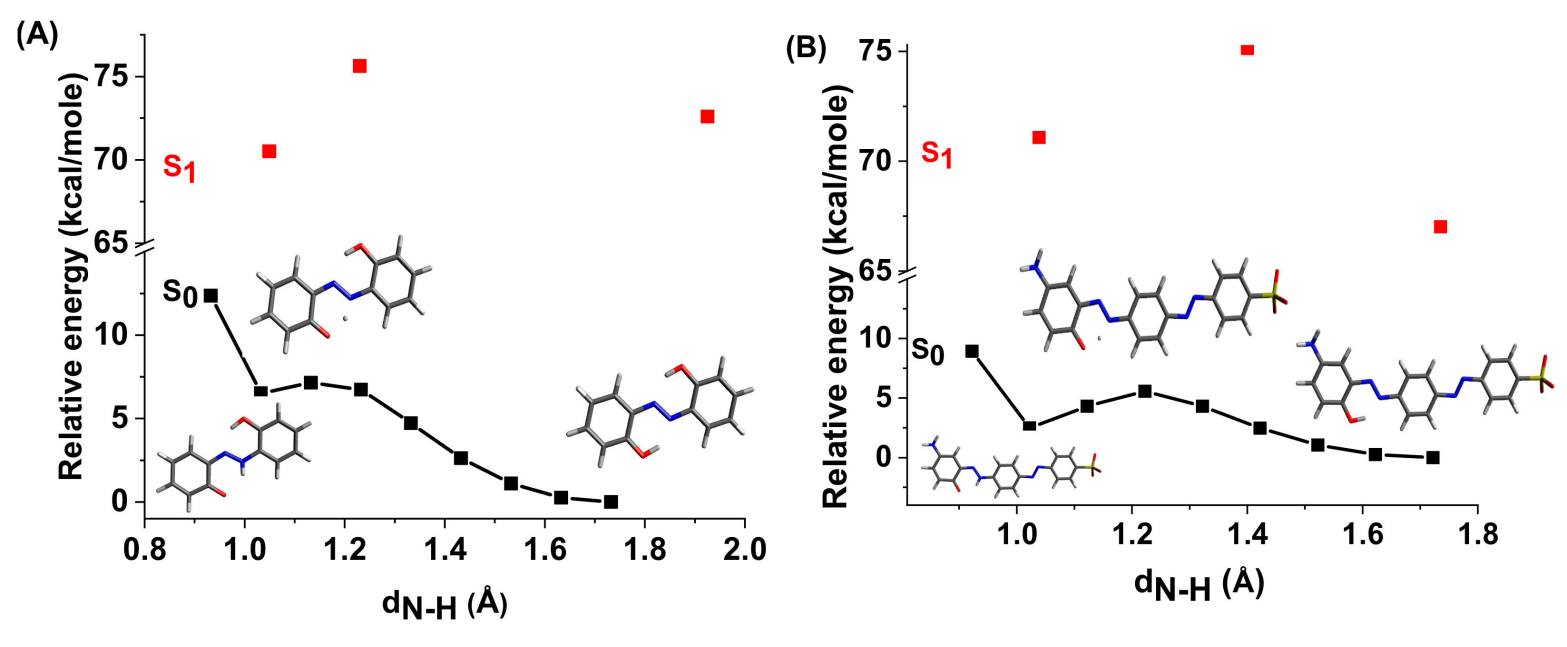
Potential energy profiles for intramolecular proton transfer in DMSO for ground and first excited states of compounds Mono_A (**A**) and Di_A (**B**).

**Figure 5:**
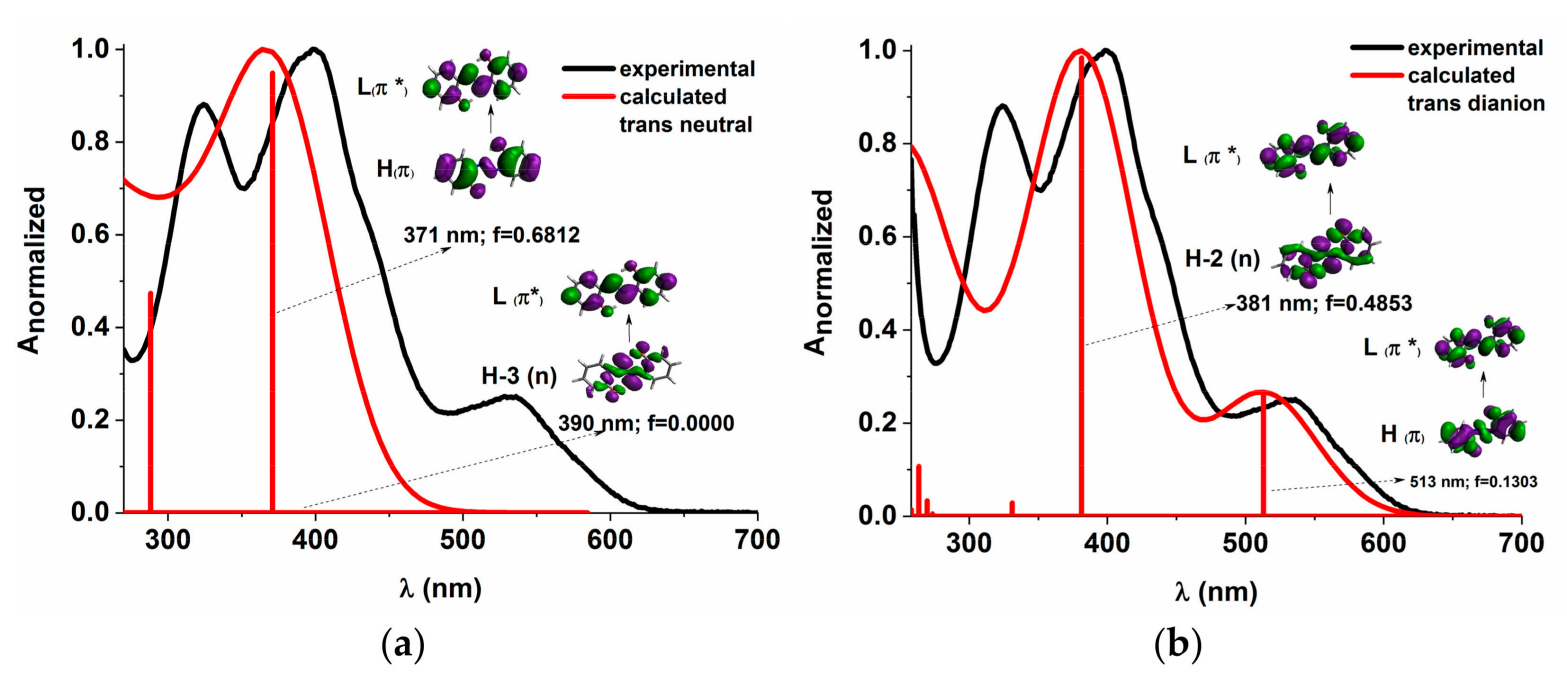
Experimental vs. calculated absorption spectra in DMSO for Mono_A neutral (**a**) and dianion (**b**). f represents the oscillator strength. The iso-density value of the molecular orbitals is 0.02 e/Bohr^3^.

**Figure 6:**
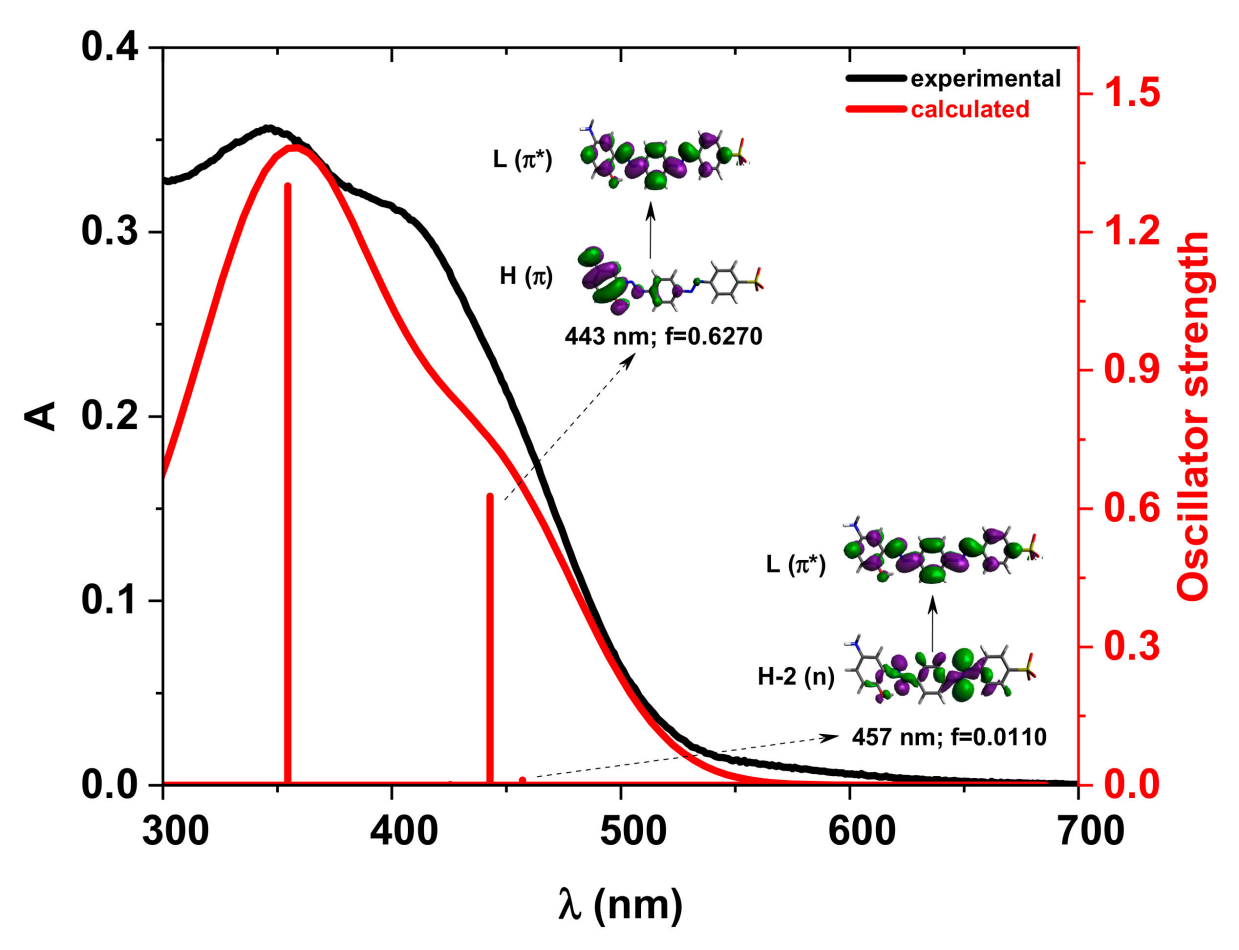
Experimental vs. calculated absorption spectra in DMSO for the compound Di_A neutral structure. f represents the oscillator strength. The iso-density value of the molecular orbitals is 0.02 e/Bohr^3^.

**Figure 7:**
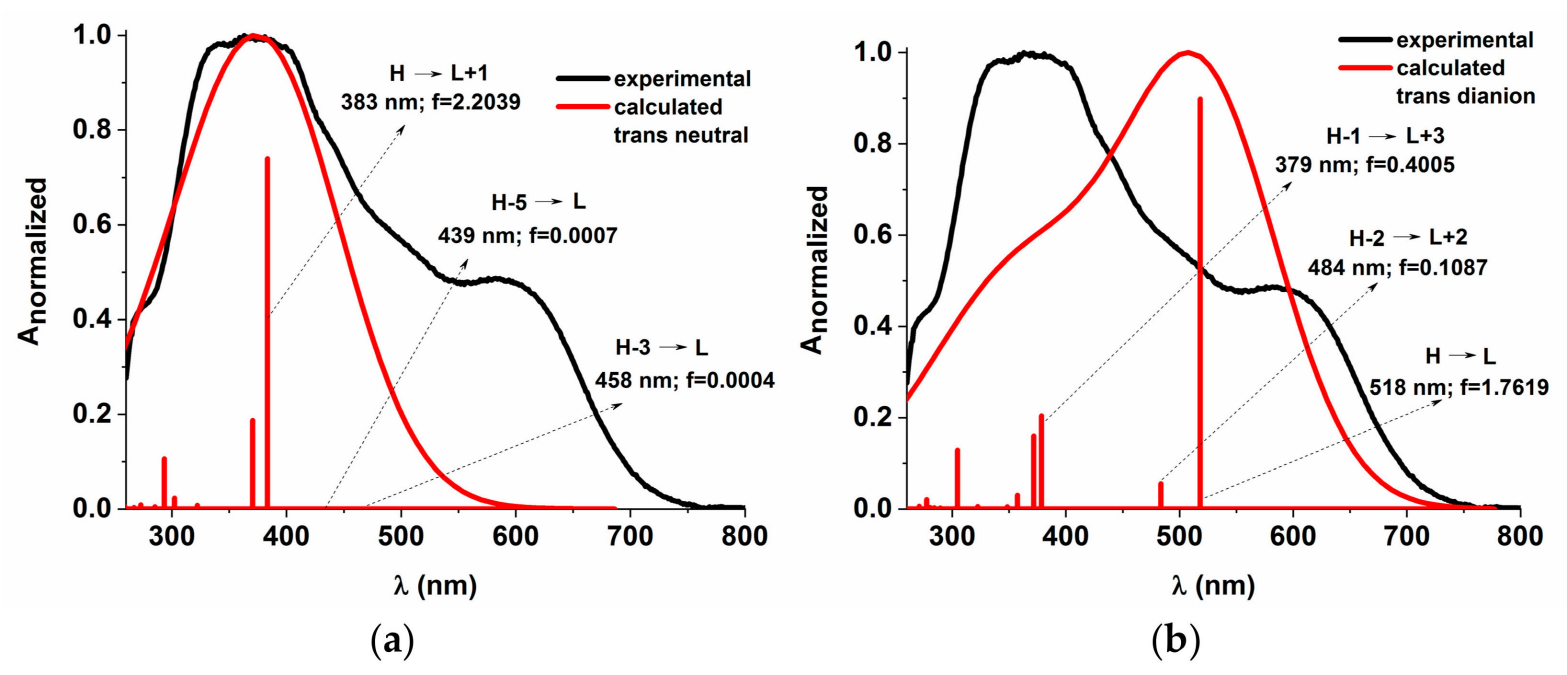
Experimental vs. calculated absorption spectra in DMSO for Tris_A derivative neutral (**a**) and dianion (**b**). f represents the oscillator strength.

The energy profiles of the intermolecular proton transfer potential for mono-azo and bis-azo in DMSO are shown in Figure 4. In the ground state, the azo form is more stable than the hydrazo form for both compounds. The energy barrier for the azo → hydrazo conversion is 7.5 kcal/mol for mono-azo and 6.5 kcal/mol for bis-azo, making intramolecular proton transfer unlikely. Therefore, the hydrazo form is not responsible for their absorption spectra. Calculated absorption spectra compared with experimental data for all azo dyes in DMSO are shown in Figure 5, Figure 6 and Figure 7. For Mono_A and Tris-A derivatives, however, neither the neutral azo structure can explain the band from 550 nm (Figure 5 and Figure 7). However, if we consider an equilibrium between neutral azo and dianion azo species, this band can be justified. The transitions involved in the lowest energy bands are also shown in Figure 5.

For the compound Di-A, the TD-DFT calculated absorption spectrum based on the neutral azo structure agrees well with the experimental one (Figure 6). The first two excitations, occurring at 457 nm and 443 nm, result from the n→π* and π→π* transitions, respectively.

The large difference between emission and absorption maxima, i.e., large Stokes shift observed for mono-azo and tris-azo, suggests an ESIPT process. In fact, calculations performed for mono-azo revealed that, in the first excited state, the energy barrier for proton transfer between −OH and −N=N- is only 2.4 kcal/mol and that the hydrazo form is more stable than the azo form (Figure 4). The emission of dianion was ruled out for both mono-azo and tri-azo since we found their theoretical emission maximum at 751 nm and 798 nm, respectively. The emission from hydrazo agrees well with the experimental value (568 nm vs. 540 nm for monoazo and 569 nm vs. 535 nm for tris-azo) and therefore it is plausible that the emitting species is the hydrazo form.

### 3.3. Spectroscopic Characterization of the Synthesized Azo Compounds

**Figure 8:**
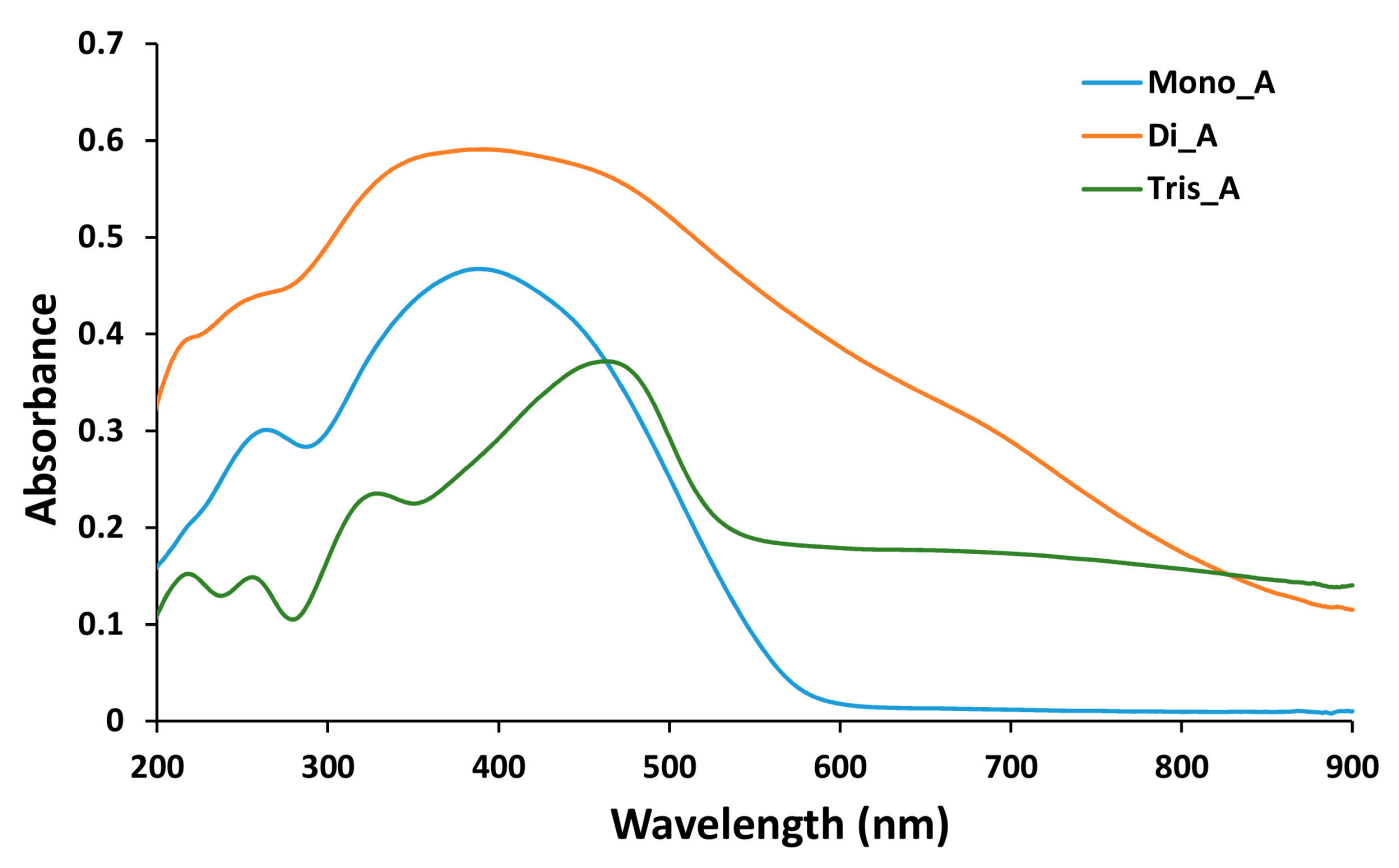
UV-Vis spectra of solid azo compounds Mono_A, Di_A and Tris_A.

In Figure 8 absorption spectra of the azo derivatives, measured in the powder, are presented.

Two absorption bands were found in the UV-Vis spectrum for (*E*)-2,2’-(diazene-1,2-diyl)diphenol Mono_A, 264 nm for the phenol moiety of the molecule and 389 nm for π_1_ →π_1_* of azo-benzene, in accordance with the literature [37]. A wider band corresponding to the two azo groups [38] in the spectrum of Di_A compound at 390 nm was attributed to the two −N=N groups, the 258 nm band for −OH phenol [39] and the 220 nm band for aniline residue [40]. The broad band at 464 nm and the band at 330 nm were attributed to the tris-azo structure substituted with the −SO_3_Na group from the spectra of compound Tris_A [41] and the bands at 257 nm and 218 nm correspond to the presence of the phenol moieties [37].

**Figure 9:**
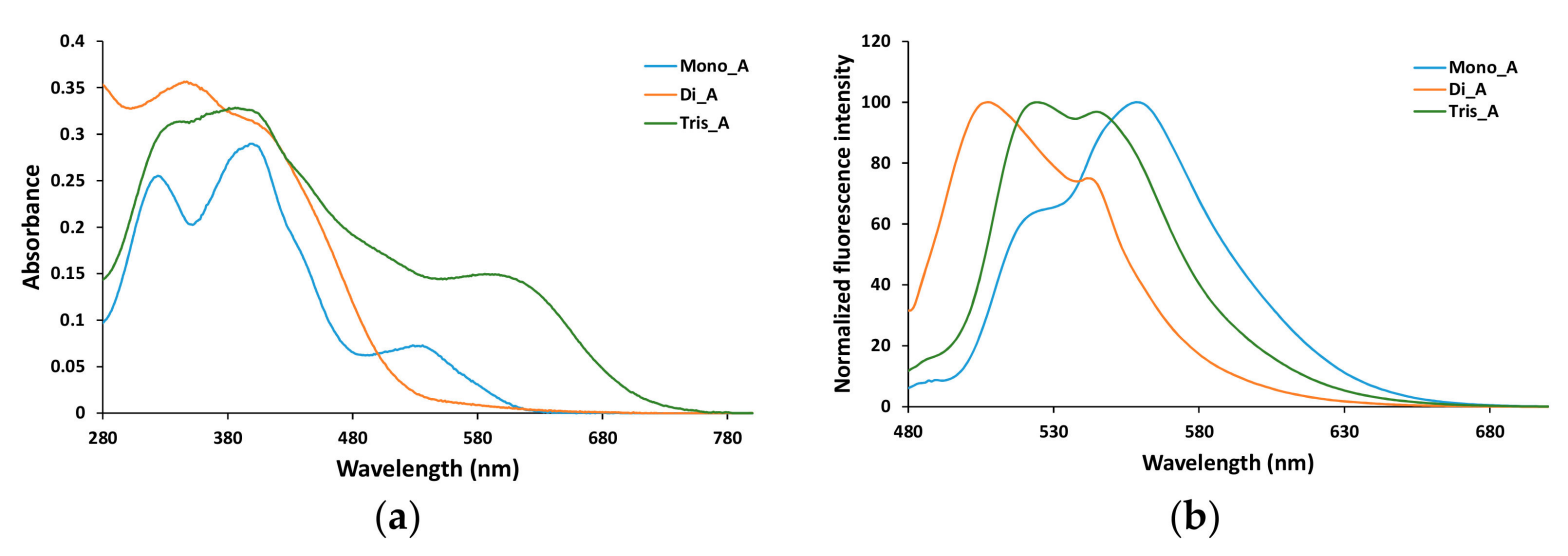
Absorption (**a**) and emission (**b**) spectra of azo derivatives in DMSO at 5 × 10^−5^ M concentration. (Excitation wavelength 400 nm).

As is suggested by the DFT results, the optical properties of the azo dyes significantly change in solution. The absorption and fluorescence spectra of the synthesized compounds are recorded in DMSO, since the highest solubility was found in this solvent for all the azo derivatives, and their absorption and fluorescence spectra are presented in Figure 9.

Broad bands in the region 300–400 nm are recorded for bis and tris azo derivatives (compounds Di_A and Tris_A), while the mono azo parent derivative exhibits two distinct peaks at 327 and 402 nm. The compounds Mono_A and Tris_A show also a shoulder located approximately at 550 nm and at 650 nm respectively (Figure 9a).

In solid state (powder) for all the three azo derivatives, no fluorescence emission was recorded under excitation at 350, 400 or 450 nm. Solubilization in DMSO leads to a development of a strong fluorescence emission, all three azo compounds showing rather broad emission peaks (Figure 9b). When excited at 400 nm, Mono_A derivative shows a maximum emission at 559 nm, with a shoulder at 525 nm. For Di_A derivative, the maximum emission is blue shifted, at 508 nm, with a small shoulder at 544 nm, while the triazo compound (Tris_A) presents two peaks at 524 and 545 nm. As is discussed in the previous section, based on the DFT results and the positions of the maximum emissions in the experimental fluorescence spectra, it is presumable that in DMSO solution the hydrazo form of the compounds is present.

**Figure 10:**
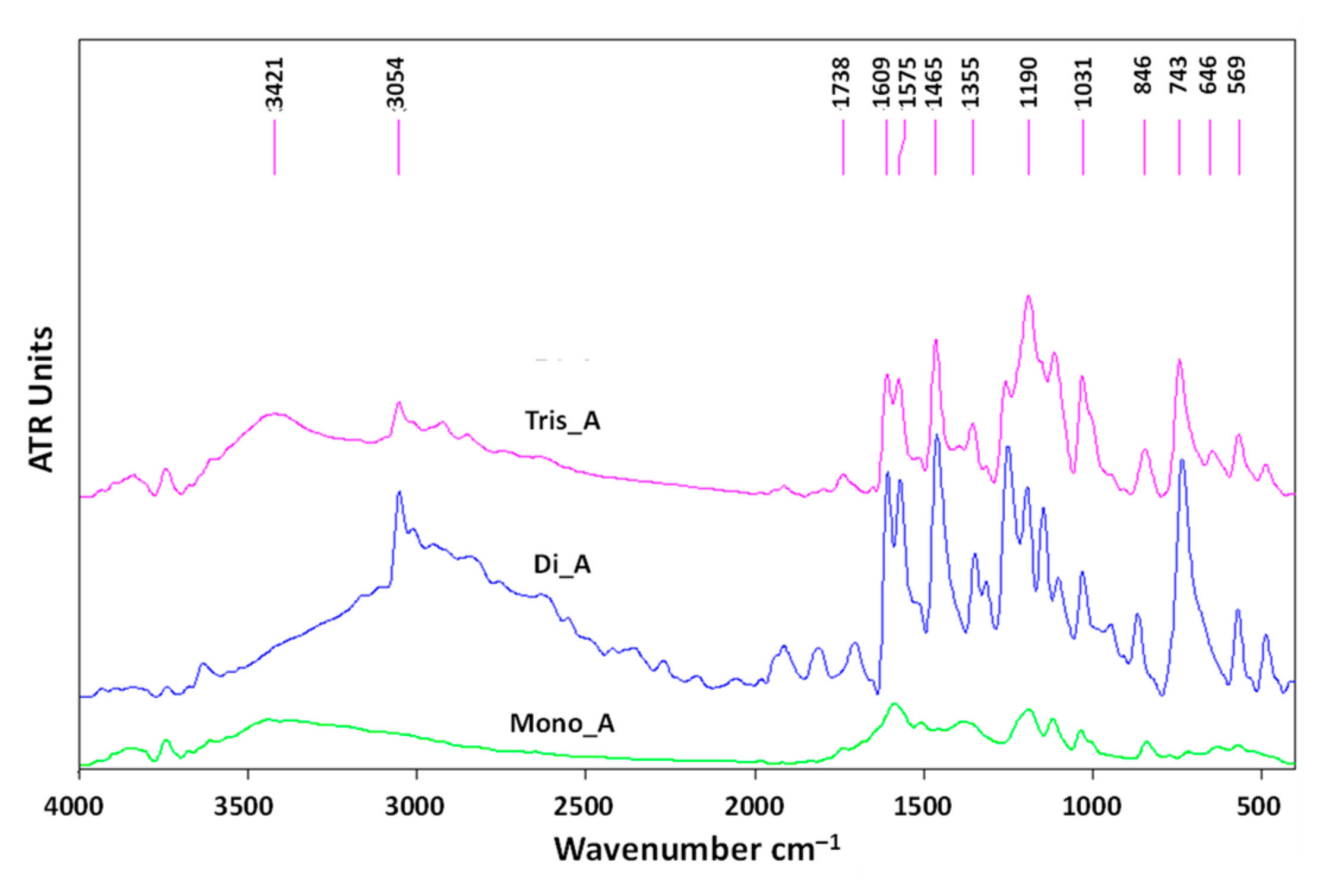
The FTIR spectra of azo derivatives.

In order to confirm the structure of the synthesized azo compounds in solid state, the FTIR spectra of mono, di and triazo derivatives were determined and are presented in Figure 10. The specific bands and their significance are listed in Table S2 in the Supplementary Materials File.

As is expected, all three derivatives exhibit sharp signals at 1607, 1588 and 1609 cm^−1^, attributed to the −N=N- group. In the FTIR spectra of the new azo-derivatives, the bands at 631 cm^−1^ (for Di_A) and 646 cm^−1^ (for Tis_A) correspond to the −SO_3_Na. All three azo compounds exhibit a specific peak at 1194 cm^−1^ (Mono_A) and 1190 cm^−1^ (Di_A and Tris_A) corresponding to the C−O band stretching phenol. The signals located at approximately 3050 cm^−1^ (at 3052 cm^−1^ for Di_A and at 3050 cm^−1^ for Tis_A) are attributed to −OH stretching. The bands at 3440 cm^−1^ present in the spectrum of Mono_A and 3421 cm^−1^ for Tris_A correspond to the aromatic −NH_2_ group.

### 3.4. Electrochemical Properties

**Figure 11:**
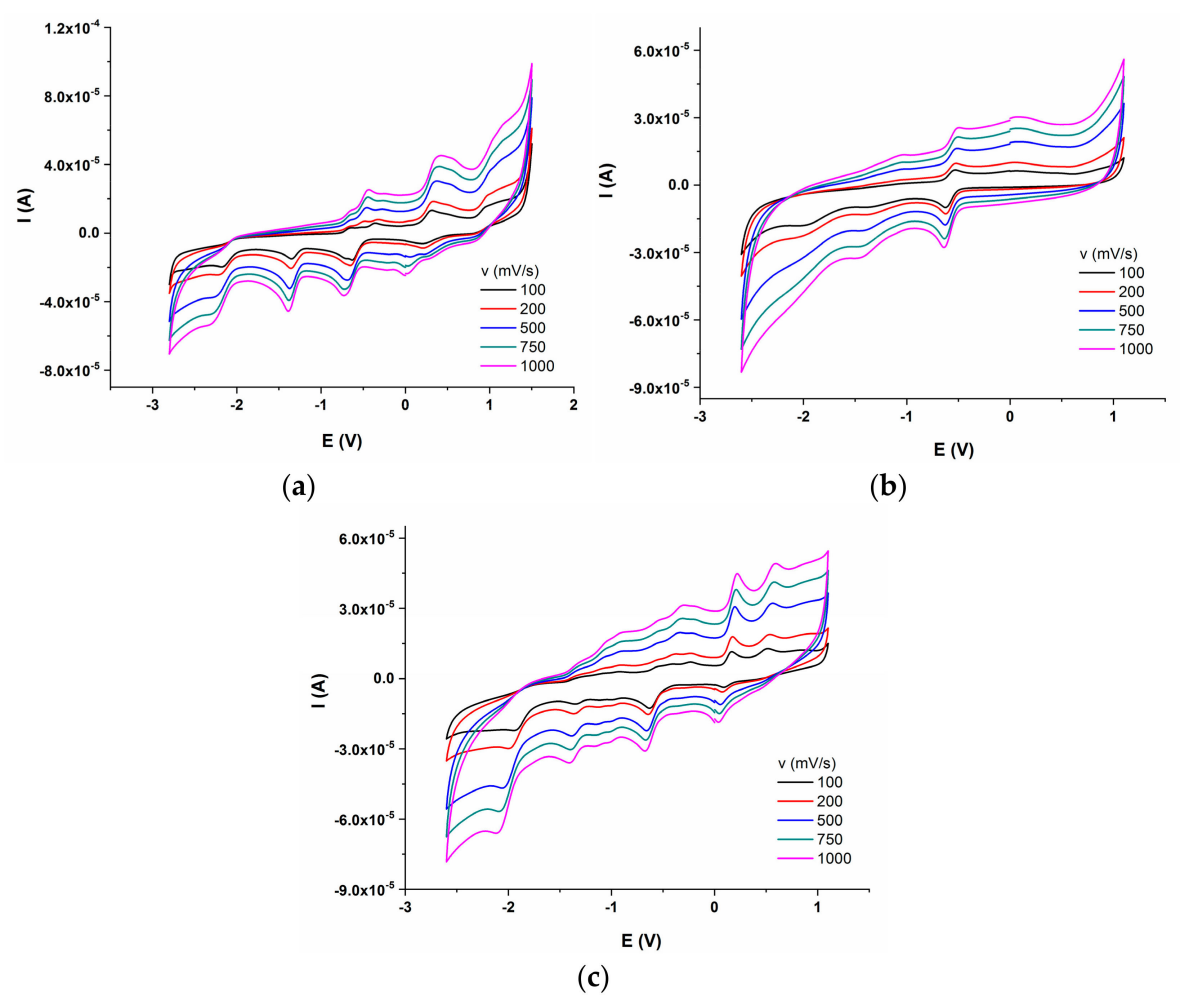
Cyclic voltammograms of azo compounds (0.001 M) in DMSO/Bu_4_NBF_4_ (0.1M); (**a**) Mono_A; (**b**) Di_A; (**c**) Tris_A.

Electrochemical behaviour of the synthesized compounds was investigated, in order to corelate their redox potential to possible in vitro and in vivo interaction with redox active species. The electrochemical properties of the three azo compounds were determined by CV and DPV measurements. The dependence of current intensity versus applied potential for the synthesized azo compounds, at different scan rates are presented in Figure 11a–c.

**Figure 12:**
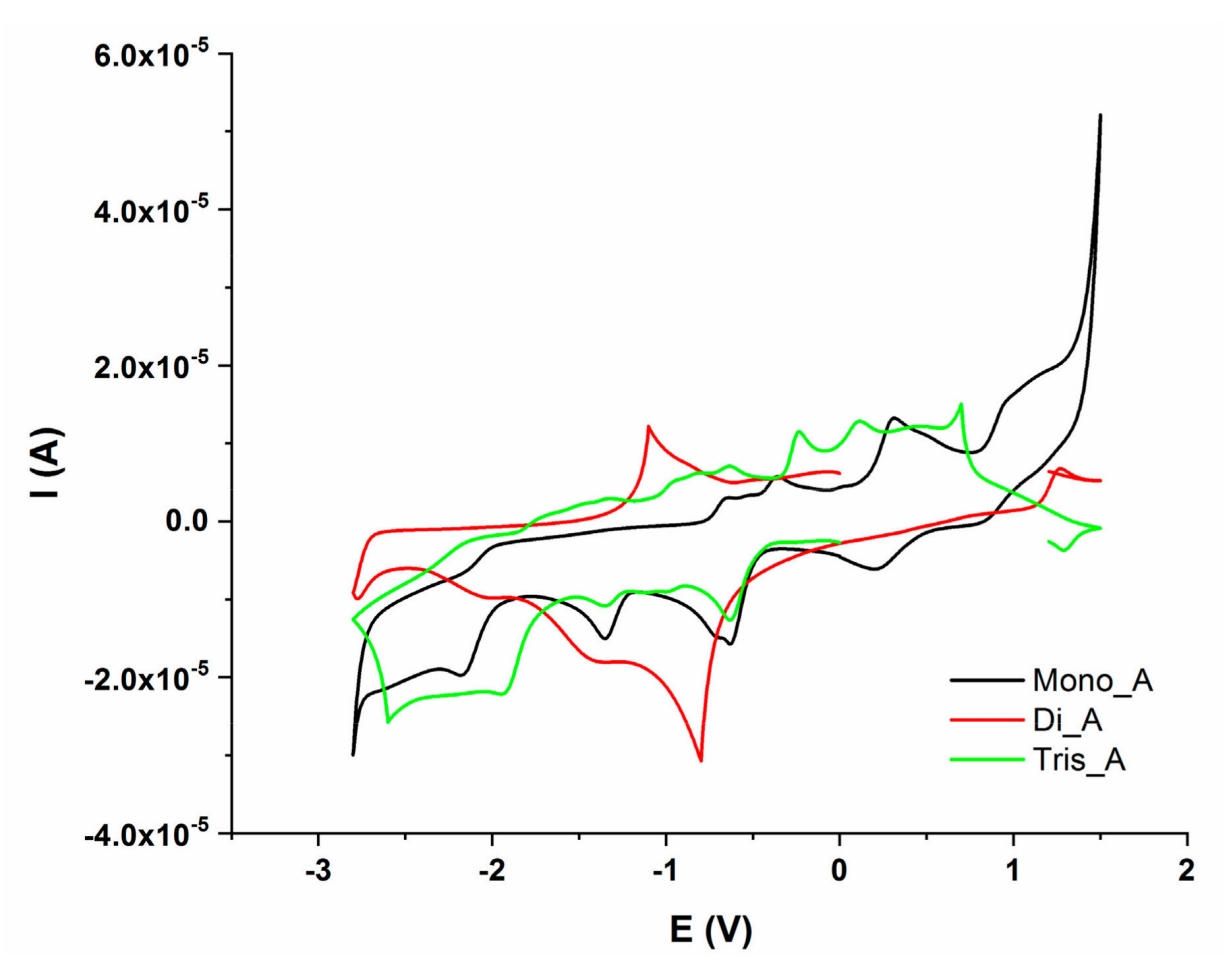
Cyclic voltammograms of Mono_A (black), Di_A(red) and Tris_A (green), potential domain (−2.8 V to + 1.5 V), scan rate 0.1 Vs^−1^.

A comparison between redox activity of the three compound at the same scan rate (100 mV/s) is presented in Figure 12. DPV measurements are presented in Figure S1 in Supplementary Materials (a—anodic trace and b—cathodic trace).

All three compounds presented electrochemical behavior. The Mono_A compound has two oxidation and five reduction peaks, compound Di_A has two oxidation and two reduction peaks and the third, compound Tris_A, showed three oxidation and four reduction processes. The redox behavior of hydrazones is influenced by the imine moiety, being a site of reduction or oxidation processes. There are several studies performed on the electrochemical behavior of azo-compounds which showed that the azo moiety is the redox active center. From previous studies, in aprotic solvents, the reduction of azo group occurs in two one-electron steps [42]. The two reduction processes are presented in all three compounds at around −1.34 V and −2 V. The oxidation peaks can be attributed to azo or aryl groups. Electro-oxidation of azo derivatives containing phenolic or amine groups can produce quinones or dimers depending on these groups’ position. The oxidation process has been observed [43]. The oxidation reaction is irreversible and often uncontrolled. For Mono_A and Tris_A compounds with a hydroxyl group in the ortho position with respect to the azo bridge, well defined irreversible oxidation peaks were recorded at around 0.3 V for M, 0.5 V for Tris_A, but were not visible for Di_A [44].

### 3.5. Antioxidant Activity

Antioxidant ability of azo synthesized compounds was examined by DPPH radical scavenging activity. The stable commercial radical is widely used as a direct indicator of antioxidant property of sample compounds. Table 2 illustrates the values of DPPH free radical scavenging of analyzed samples expressed as caffeic acid equivalents (CAE, μg/mL) and gallic acid equivalents (GAE, μg/mL).

It is found that compounds Mono_A and Tris_A showed a somewhat stronger antioxidant activity than compound Di_A compared to both considered references antioxidants CAE and GAE, but with moderate values compared to other azo compounds reported in the literature [45,46].

Antioxidant capacity is an electron/proton donating capacity of the compound, which depends on the presence of hydroxyl groups in the ortho position (two for Mono_A and Tris_A, one for Di_A) in the phenolic ring, and does not depend on the presence of the amino group in the meta position (for Di_A) [47]. Consequently, the phenolic compounds Mono_A and Tris_A exhibited a better antioxidant activity, which can be favored by the structure and azo extended π electron conjugation of the compound, and several azo-hydrazo tautomeric structures in these cases.

### 3.6. Interaction with Serum Protein

The interaction of drug with serum protein is of particular interest, because by binding to biomolecules many active compounds decrease their therapeutic efficiency. BSA, often used as model serum protein, exhibits a strong fluorescence produced by the tryptophan (Trp), tyrosine (Tyr) and phenylalanine (Phe) residues; for the latter, a lower contribution is involved. The binding of drug molecules results in changes in Trp and Tyr residues’ microenvironment and it is noticed as fluorescence quenching [48].

**Figure 13:**
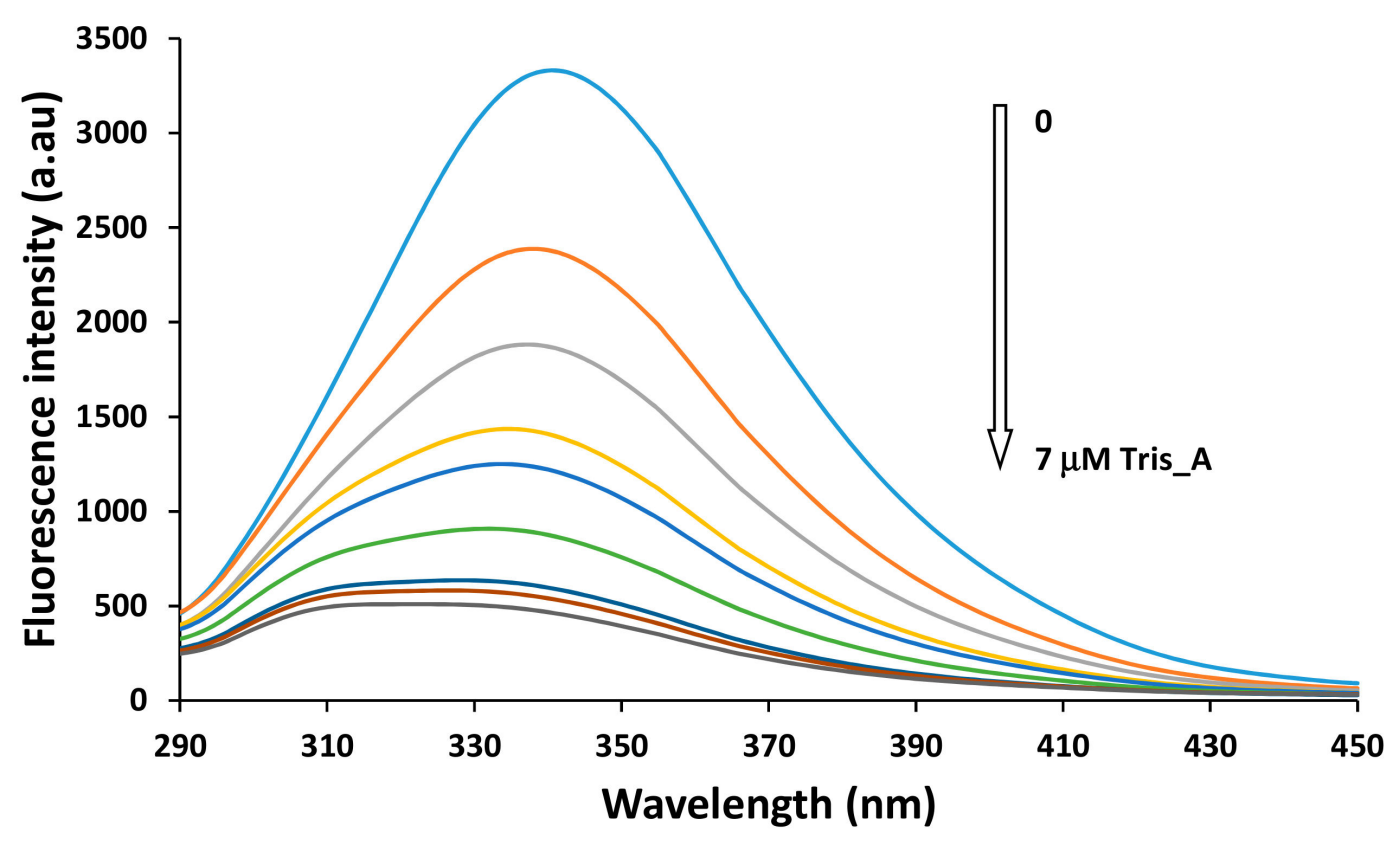
Fluorescence spectra of BSA (2 μM) in the absence and presence of various concentration of azo compounds Tris_Azo at 298 K. (Concentrations of the quencher are in the range 0÷7 μM).

In Figure 13, the variation of the fluorescence intensity of the BSA solution in the presence of the Tris_A azo derivative is shown as representative behavior. For the other two azo compounds the fluorescence variations are included in the Supplementary Materials File.

For all three compounds, the increase in their concentration in the solution of BSA leads to a sequentially decrease of the fluorescence intensity of the protein. A significant blue shift of the maximum emission is also observed, from 340 nm up to 325 nm, in the case of compound Tris_A, and similar Δλ approximately 10–15 nm for Mono_A and Di_A, respectively. This is due to the changes in the hydrophobicity of the Tyr microenvironment during the binding of azo dyes on the BSA molecules [49].

In order to determine the binding mechanism, the fluorescence quenching data are analyzed using the Stern-Volmer equation [30]:

(2) $$F_{0}/F=1+K_{SV}\cdot \left[Q\right]$$

where *F_0_* and *F* are the corrected emission intensities of the protein in the absence and in the presence of quencher and *Q* is the concentration of the quencher species, respectively.

**Figure 14:**
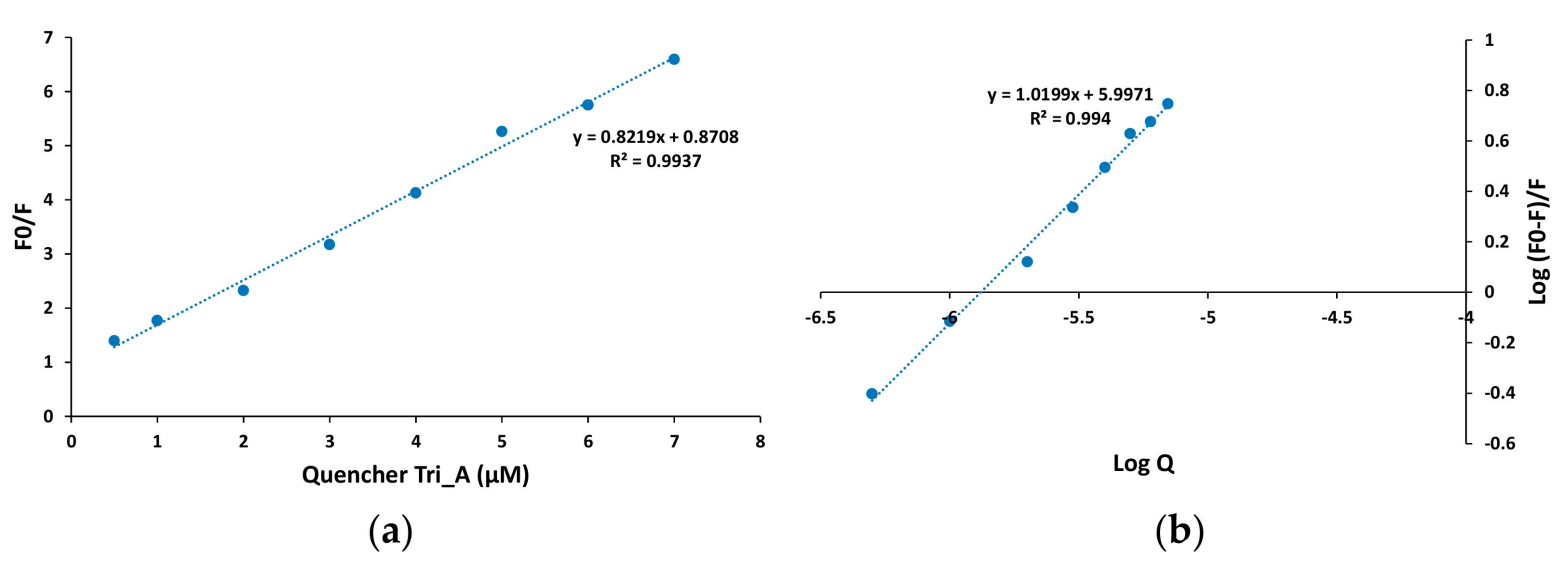
Stern-Volmer plot (**a**) and double logarithmic plot (**b**) for fluorescence quenching in the BSA-azo derivative Tris_A solutions at 298 K.

As is shown in Figure 14 for Tris_A derivative, the Stern-Volmer plot of BSA at lower concentrations, up to 10 µM of azo compound, is linear, which suggests a static quenching process.

The Stern-Volmer plots for Mono_A and Di_A azo compounds also exhibit high linearity (Figures S3 and S4), thus it is presumable that for all new synthesized derivatives a static quenching mechanism is involved. The values of the K_sv_, the Stern-Volmer quenching constant, were found to be in the range of 1–7.9 × 10^5^ M^−1^, with increasing value from Mono_A derivative (1.01 × 10^5^ M^−1^) to 1.83 × 10^5^ M^−1^ for Di_A and to the highest value of 7.96 × 10^5^ M^−1^ for Tris_A.

Considering that the quenching process is static and occurs with quenching molecules binding independently to a number of equivalent sites on the protein, the binding constant *K_b_* and the number *n* of binding sites were determined. The strong affinity to BSA could result in a longer half-life in the body, thus the binding characteristics provide information about the pharmacokinetics of the compound. The binding parameters (*K_b_* and *n*) were calculated using the double logarithmic equation [30]:

(3) $$\log\left(\left(F_{0}-F\right)/F\right)=\log K_{b}+n\cdot \log\left[Q\right]$$

where *F*_0_, *F* and *Q* have the same significance as that in Equation (2). The double logarithmic plot for Tris_A as representative azo dye derivative is shown in Figure 14b. The plots for Mono_A and Di_A azo compounds are presented in Figures S3 and S4.

The values of 10^4^–10^5^ M^−1^ obtained for the binding constants suggest a moderate interaction between the azo derivatives and BSA molecules. The binding constants of Mono_A and Di_A are similar, 6.44 × 10^4^ and 5.14 × 10^4^ M^−1^, respectively, while for Tris_A derivative a higher value of 9.93 × 10^5^ M^−1^ is obtained.

The number n was found to be 1.02 for Tristan, 0.89 for Di_A and 0.96 for Mono_A, thus, one can conclude that the azo compound-BSA association is produced in 1:1 ratio [50]. Our results are similar to the behavior reported for other azo derivatives [51].

### 3.7. Predicted Pharmacokinetics and Drug Likeness Properties

To evaluate the new synthesized azo dyes as potential drug candidates, their pharmacokinetic and drug-likeness parameters were computed using the SwissADME online tool. The results of ADME analysis for mono-, di- and tris-azo derivatives are presented in Table S4 in Supplementary Materials File.

Both tautomeric forms were investigated for all the azo derivatives. The changes in the chemical structure at the azo bond leads to differences in the topological polar surface area (TPSA), with lower values for the hydrazo form. Both hydrazo and azo forms of the Mono_A derivative possesses satisfactory values (61.69 Å^2^ and 65.18 Å^2^) of TPSA to fulfill the Veber rule (TPSA < 140 Å^2^). In the case of derivative Di_A, the hydrazo form show a TPSA value of 143.95 Å^2^, close to the maximum admissible, but the other tautomer have a significantly higher value (181.68 Å^2^). As it is expected, Tris_A derivative, both forms are far from the required TPSA threshold, with calculated values 162.88 Å^2^ and 200.61 Å^2^, respectively. Thus, it is assumed that the Tris_A compound will have a low oral bioavailability, while the Di_A derivative, and especially the Mono_A derivative, will have a higher oral absorption bioavailability.

The lipophilicity was evaluated from logP values, computed as the average of the five results obtained with the different models included in the SwissADME tool. The obtained values for the studied azo derivatives are 2.72 in the case of Mono_A (1.56 for hydrazo form), relatively close values in the case of Di_A compound (2.93 and 1.56), and higher ones for the most lipophilic Tris_A (4.59 and 3.24, respectively). The gastrointestinal absorption and brain penetration properties are very important pharmacokinetic characteristics to be evaluated in the drug development procedure. Mono-azo derivative shows high gastrointestinal absorption (GI) and blood-brain barrier (BBB) permeation in both tautomeric forms, while the compounds Di_A and Tris_A possess low GI and do not penetrate BBB.

Lipinski’s rule (rule of five) recommends for a drug candidate no more than one violation. As is predicted based on ADME parameters, compounds Mono_A and Di_A pass the Lipinski’s rule with no violation, while the Tris_A compound exhibits two violations, due to the high molecular weight and the number of N and O atoms. Since the tri-azo derivative is not compliant with neither Lipinki nor Veber rule (due to the large value of TPSA), the most promising candidate as drug from the novel azo synthesized is the Di_A compound.

### 3.8. Antifungal Activity

The antimicrobial activity of the new azo derivatives was tested against some fungal *Candida* strains, both standard and clinically isolated. The results of the inhibition zone diameters are listed in the Table 3.

From the qualitative evaluation, a standard concentration of 1 mg/mL of azo derivative shows no inhibition of the fungal growth in the case of Di_A, while for Mono_A and Tris_A the values of the inhibition zone diameters are close or slightly smaller than the reference drug Fluconazole for *Candida albicans* ATCC 10231 and *Candida pasapsilosis* CL 5348. However, both of the azo derivatives are efficient against clinical isolates strains *Candida tropicalis* CL 3301, *Candida krusei* CL 5343 and *Candida albicans* CL 7626.

**Figure 15:**
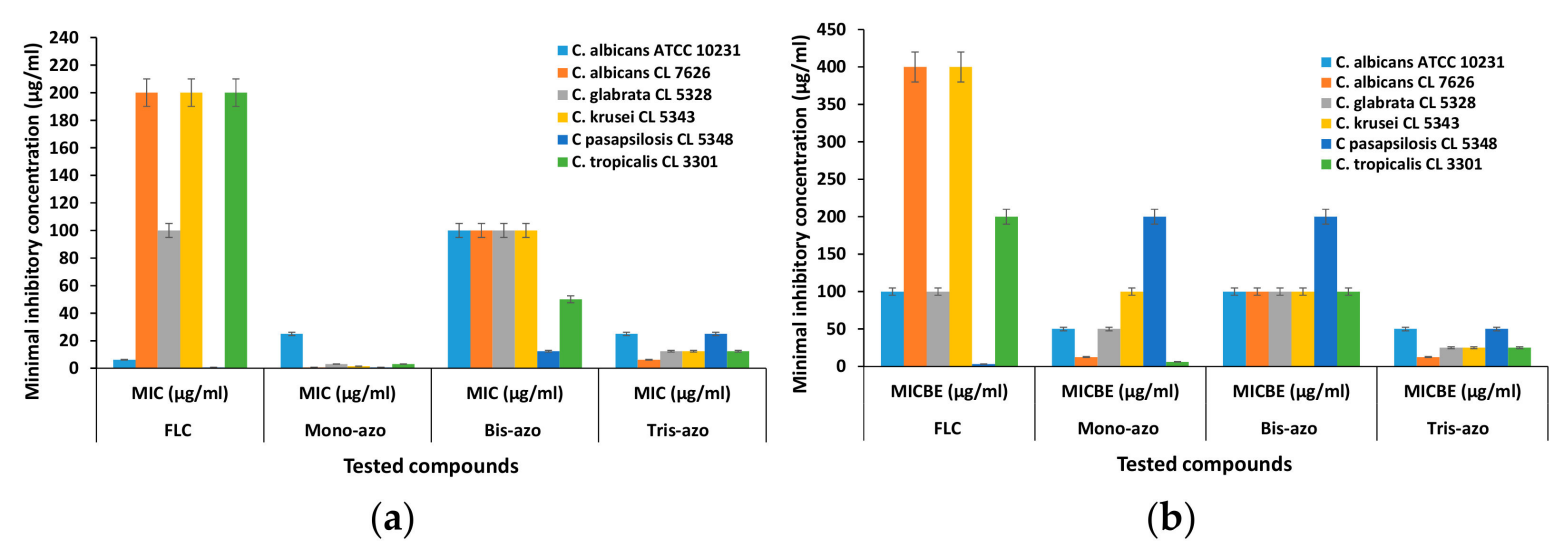
Graphic representation of Minimal inhibitory concentration (MIC) (**a**) and Minimal inhibitory concentration for biofilm eradication (MBEC) values (**b**) for the azo derivatives.

The antifungal activity was quantified using fungi strains both in planktonic growth, expressed as minimum inhibitory concentration (MIC), and against respective biofilms formed, expressed as minimum biofilm eradication concentration (MBEC). The results regarding the MIC and MBEC values of the new di- and tri-azo derivative compared to mono-azo and Fluconazone as reference drug are comparatively represented in Figure 15.

The lowest MIC values were obtained for the mono-azo compound (between 0.781 and 25 µg/ml), followed by the tris-azo MIC values (between 12.5 and 25 µg/ml), which confirms the qualitative results. Generally, Di_A derivative exhibits the lowest efficiency among the new derivatives, but compared to reference Fluconazole shows smaller values of MIC for the clinical strains *C. albicans* CL 7626, *C. glabrata* CL 5328, *C. krusei* CL 5343 and *C. tropicalis* CL 3301.

The microorganisms cultivated on a surface where they are able to organize in biofilms show an increase in resistance, compared to the floating cells grown in suspension. Thus, it is very interesting that the new synthesized azo compounds possess much lower MBEC values compared to the reference drug Fluconazole for the majority of the tested *Candida* strains, including Di_A derivative.

Additionally, the tris-azo compound Tis_A significantly inhibited the adherence capacity of all tested strains, expressing the lowest MBEC values, probably because of its highest hydrophobicity.

A peculiar behavior is observed with *Candida pasapsilosis* CL 5348, which is a commensal of human skin with low pathogenicity for an immunocompetent host. This *Candida* specie is recognized as a very versatile species regarding its capability to adhere and to form biofilms on catheters and other implanted plastics and for persistence in the hospital environment [52]. Its virulence property appears associated with a capacity to produce phospholipase and aspartyl protease [53] and could explain the behavior of *Candida pasapsilosis* CL 5348 strain in the presence of antimicrobial compounds with different chemical composition, along with higher MBEC values.

Even if *Candida pasapsilosis* CL 5348 strain expressed the lowest MIC values when exposed to all tested compounds, including fluconazole, the MBEC values were the lowest for fluconazole, but much higher for mono and bis-azo compounds, compared to the other strains.

As a general observation, the new bis- and tris-azo derivatives (Di_A and Tris_A) exhibit a significantly increased efficiency compared to Fluconazole antifungal drug, in particular against biofilms formed by clinically isolated strains of *Candida*, with the exception of *Candida pasapsilosis* CL 5348, which seems to be more sensitive to treatment with Fluconazole.

In Table 4, a series of properties of azo-chromophores have been summarized, in order to highlight the similarities and differences between those synthesized in the present work and those reported in the literature.

## 4. Conclusions

In conclusion, two novel (phenyl-diazenyl)phenol based azo dyes, containing two and three –N=N- bonds, were obtained, using a simple, convenient and energy saving synthesis. The DFT study reveals the possible existence of azo and hydrazo tautomeric forms, and computed absorbance and fluorescence spectra are discussed, compared to the experimental ones. The hyperpolarizability (β) and its components computed show very high values for Di_A (bis azo) and Tris_A (tri azo) derivatives, which suggests superior NLO properties. The new synthesized azo compounds and the mono azo precursor show no significant antibacterial activity, while the efficiency against various *Candida* strains is very good, in some cases better than the effect of Fluconazole used as reference antifungal drug. Mono_A and Tris_A derivatives show significant inhibition effect on both microorganisms cultivated in solutions and on the biofilm development of some clinically isolated strain of *Candida* (*C. albicans* CL 7626, *C. glabrata* CL 5328, *C. krusei* CL 5343 and *C. tropicalis* CL 3301). All the studied azo dyes possess the ability to bind to serum albumin, with a stronger tendency exhibited by Tris_A derivative, as the most hydrophobic in nature. 

Based on the results obtained for optical, electrochemical and antifungal properties, the novel azo dyes could be further optimized, in order to become valuable compounds to be applied as NLO materials, antioxidants in the food industry or antifungal agents.

## Figures and Tables

**Table 1:** Energy difference between frontier orbitals Δ_EH-L_, mean isotropic polarizability α, anisotropy of polarizability Δα (10^−23^ e.s.u.), dipole moment μ, and first static hyperpolarizability β values determined from DFT (CAM-B3LYP/6-311++G**) calculations in vacuum.

Substance	ΔE_H-L_ (eV)	α (10^−23^ e.s.u.)	Δα	μ (D)	β (10^−30^ e.s.u.)
Mono_A	5.89	2.86	3.11	0.00	0.0005
Di_A	4.24	5.97	8.16	33.53	184.73
Tris_A	3.66	7.83	10.09	46.12	199.51

**Table 2:** The values of DPPH free radical scavenging of analyzed samples.

Sample	Concentration (μg/mL)	
	CAE ^1^	GAE ^2^
Mono_A	6.99 ± 0.53 ^3^	3.40 ± 0.26
Di_A	6.86 ± 0.40	3.37 ± 0.20
Tris_A	6.99 ± 0.53 ^3^	3.40 ± 0.26

^1^ CAE: caffeic acid equivalents; ^2^ GAE: gallic acid equivalents; ^3^ standard deviation, (n = 3 determinations).

**Table 3:** Results regarding the inhibition zone diameters expressed by the tested compound in the contact with the yeast strains’ cells.

Tested Strains	Inhibition Zone Diameters (mm)			
	FLC (1 mg/mL)	Mono_A (1 mg/mL)	Di_A (1 mg/mL)	Tris_A (1 mg/mL)
*Candida pasapsilosis* CL 5348	18	10	0	5
*Candida tropicalis* CL 3301	0	10	0	8
*Candida glabrata* CL 5328	0	0	0	7
*Candida krusei* CL 5343	0	13.5	0	6
*Candida albicans* CL 7626	0	9	0	10
*Candida albicans* ATCC 10231	18.5	18.5	0	10

**Table 4:** A comparison of the novel azo dyes with relevant other compounds reported in literature.

No	Synthesis and Properties	In This Paper	In Other Papers
1	Synthesis of azo π push-pull chromophores	Classic syntheses—diazotization and coupling reactions	[5,6,9,11,17,18,21,22,37,45,46]
2	HOMO-LUMOgap (eV)	3.66–5.89	2.089–2.159 [54]
			2.448–2.799 [11]
			2.02–2.38 [17]
			3.21–3.56 [21]
3	β (10−30 e.s.u.)	184.73, 199.51	202–260 [54]
4	μβ (10−48 e.s.u.)	6194, 9201	1450–4700 [55]
5	Antioxidant properties	All compounds show antioxidant activities	[45,46]
6	Antifungal properties	fungal species (*C. pasapsilosis, C. tropicalis, C. glabrata, C. krusei, C. albicans*)	fungal species (*A. niger, F. oxysporium, P. italicum* and *A. alternata*) [22]
7	Antibacterial properties	–	against *S. aureus, B. cereus, E. coli*, and *K. pneumonia* [22]
			*B. subtilis*, *S. aureus* [45]
8	Applications	Material with NLO, antioxidant and antifungal properties	dyeing of nylon and polyester; antimicrobial [56]
			NLO properties [7,17,18,55]
			NLO properties for laser applications [21]
			solar cells [11]
			antimicrobial activity and DNA binding of supramolecular [22]
			antioxidant, antibacterial and anticancer [45]
			antioxidant and anti-inflammatory [46]

## Data Availability

Not applicable.

## Supplementary Materials

The following supporting information can be downloaded at: https://www.mdpi.com/article/10.3390/ma15228162/s1, Figure S1: DPV traces of M, B and T (0.001 M) in DMSO/Bu_4_NBF_4_ (0.1 M), potential domain (a) (0.0 V–+ 1.5 V) anodic trace, (b) (0.0 V–+ 1.5 V) cathodic trace. DPV parameters were: SP=10 mV and MA=25 mV; Figure S2: Quenching of the BSA fluorescence (BSA 2 μM) in the absence and presence of various concentration of azo compounds: Mono_A (a) and Di_A (b) at 298 K. Concentrations of the quencher are in the range 0–7 μM.; Figure S3: Stern-Volmer plot (a) and double logarithmic plot (b) for fluorescence quenching in the BSA-azo derivative Mono_A solutions at 298 K. Figure S4: Stern-Volmer plot (a) and double logarithmic plot (b) for fluorescence quenching in the BSA-azo derivative Di_A solutions at 298 K. Figure S5: Inhibition zone aspect, after 24 h of yeast strains incubation, in the contact with tested compounds, for *C. albicans* CL 7626 (a), *C. albicans* ATCC 10231 (b) and *C. krusei* CL 5343 (c). Table S1: Calculated distances and angles in optimized molecules of synthesized azo derivatives; Table S2: Peak positions and their interpretation in FTIR spectra of the azo derivatives; Table S3: The equations of calibration curves, correlation coefficients and linearity domanins of obtained curves for antioxidant activity; Table S4: Pharmacokinetics and drug-likeness parameters of synthesized azo derivatives (computation using SwissADME online tools).
