# Supplementary material for: Synthesis, Characterization, DFT Study and Antifungal Activities of Some Novel 2-(Phenyldiazenyl)phenol Based Azo Dyes
Source: Materials (Basel). 2022 Nov 17;15(22):8162. doi: 10.3390/ma15228162 (PMC9695727; doi:10.3390/ma15228162)
Supplement: Supplementary file 1 [file materials-15-08162-s001.zip › materials-2022736-supplementary.pdf]

Supplementary Materials

Table S1. Calculated distances and angles in optimized molecules of synthesized azo derivatives.

Atoms	Distance (Å)	Atoms	Bond angle (deg)	Atoms	Dihedral angle (deg)
**Mono_A**					
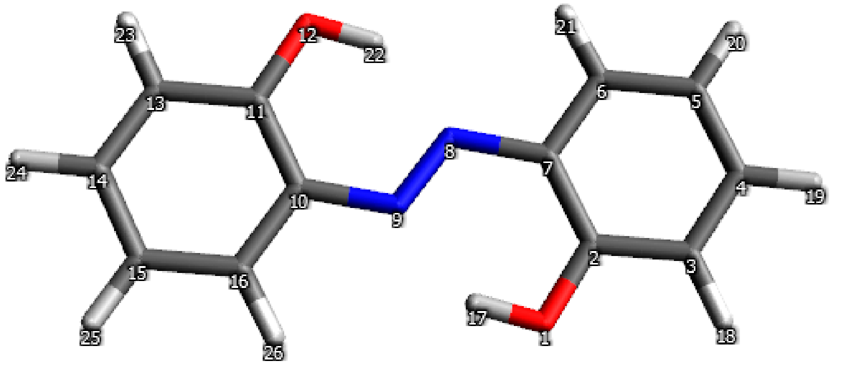					
N8-N9	1.257	N8- N9- C10	117.6	C7-N8-N9-C10	180.0
C9-N10	1.398	N9- C10-C11	124.7	N8- N9- C10-C11	0.0
C10-C11	1.409	C10-C11-O12	122.5	N9-C10-C11-O12	0.0
C11-O12	1.337	C11-O12-H22	107.1	C10-C11-O12-H22	0.0
O12-H22	0.983				
H22-H8	1.744				
H24-H19	11.198				
**Di_A**					
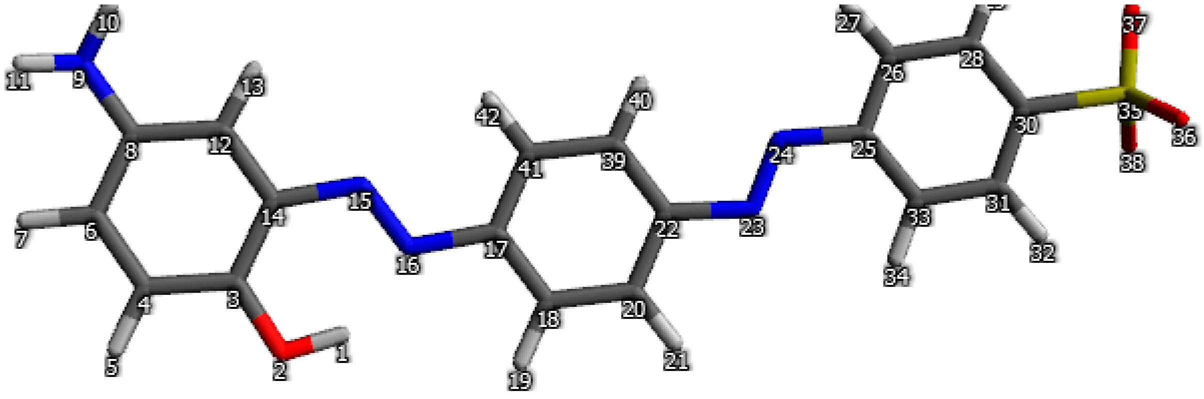					
C17-N16	1.412	C17- N16- N15	116.5	C17- N16-N15-C14	-179.9
N16-N15	1.249	N15-C14-C3	125.0	N15-C14-C3-O2	-0.2
N15-C14	1.401	C3-O2-H1	106.3	C14-C3-O2-H1	0.0
C3-O2	1.343	C25-N24-N23	116.5	C25-N24-N23-C22	179.9
O2-H1	0.968	C11-N9-C8	113.8	N23-C22-C20-H21	-0.1
H1-N5	1.735	C30-S35-O36	103.3	C11-N9-C8-C6	32.0
N23-N24	1.245			C31-C30-S35-O37	178.9
C25-N24	1.404				
C22-N23	1.417				
C11-N9	1.007				
N9-C8	1.407				
S35-C30	1.806				
S35-O36	1.460				
H29-O24	18.522				

Tris-A

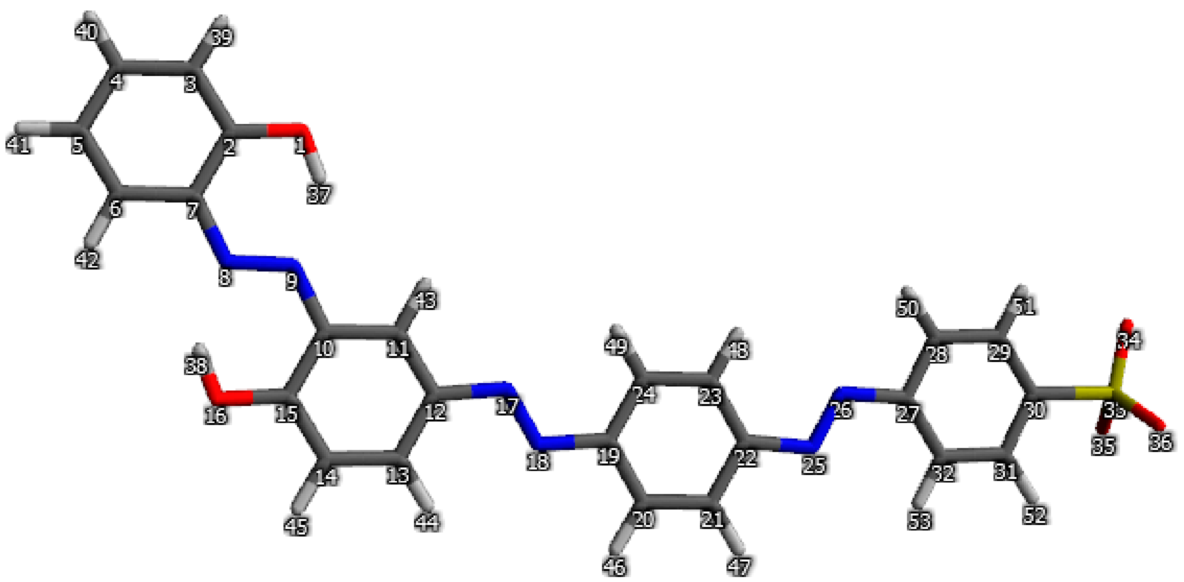

N18-N19	1.241	C27-N26-N25	116.5	C27-N26-N25-C22	180.0
N17-C12	1.416	C19-N18-N17	115.9	N26-N25-C22-C23	0.1
N18-C19	1.413	C10-N9-N8	117.6	C19-N18-N17-C12	180.0
N8-N9	1.257	O34-S33-O36	114.8	N18-N17-C12-C13	0.0
C7-N8	1.399			C10-N9-N8-C7	-180.0
N10-C9	1.397			N9-N8-C7-C2	0.0
C2-O1	1.337			C7-C2- O1-H37	0.0
O1-H37	0.983			C10-C15- O16-H38	0.0
N9-H37	1.749				
N25-N26	1.245				
C22-N25	1.418				
N26-C27	1.405				
S20-O23	1.460				
C30-S33	1.806				
H39-O22	23.038				

Table S2. Peak positions and their interpretation in FTIR spectra of the azo derivatives.

**Mono_A**		**Di_A**		**Tris_A**	
Peak (cm^{-1})	***Bond type***	***Peak (cm^{-1})***	***Bond type***	***Peak (cm^{-1})***	***Bond type***
		3440	–NH_2 aromatic	3421	–NH_2 aromatic
3052	–OH stretching		–OH stretching	3050	–OH stretching
1914	Aromatic combination bands				
1811					
1704				1739	Aromatic
1607	–N=N–	1588	–N=N–	1609	–N=N–
1571 split	–N=N–			1575 split	–N=N–
1461	C=C–C aromatic ring stretch	1508	C=C–C aromatic ring stretch	1465	C=C–C aromatic ring stretch
1349 and 1315 split	–OH bend phenol	1387	–OH bend phenol	1355	–OH bend phenol
1194	C–O stretching phenol	1190	C–O stretching phenol	1190	C–O stretching phenol
1147	Aromatic C-H out-of-plane bend				
1102	Aromatic C-H out-of-plane bend	1119	Aromatic C-H out-of-plane bend	1114	Aromatic C-H out-of-plane bend
1031	Aromatic C-H out-of-plane bend	1036	Aromatic C-H out-of-plane bend	1031	Aromatic C-H out-of-plane bend
868	Aromatic C-H out-of-plane bend	841	1,4-disubstituted benzene	846	1,4-disubstituted benzene
735	1,2-disubstituted benzene	717	mono-substituted benzene	743	1,2-disubstituted benzene
571	O-H out-of-plane bend	571	O-H out-of-plane bend	569	O-H out-of-plane bend
		631	–SO_3Na	646	–SO_3Na

Table S3. The equations of calibration curves, correlation coefficients and linearity domanins of obtained curves in antioxidant study.

**Standard**	**Equation**	**r^2 / n** [1]	**LD (µg/mL)** [2]
Caffeic acid	y [3] = 5.528x [4] + 49.66	0.9951 / 9	0.0293 - 7.50
Gallic acid	y = 11.14x + 50.03	0.9939 / 8	0.0293 - 3.75

[1] r: correlation coefficient / n:number of determinations; [2] linearity domanin;
[3] inhibition percent (% I); [4] standard concentration (µg/mL)

Table S4. Pharmacokinetics and drug-likeness parameters of synthesized azo derivatives (computation using SwissADME online tools).

Molecule	Mono_A NH	Mono_A OH	Bis_A NH	Bis_A OH	Tris_A NH	Tris_A OH
Num. heavy atoms	16	16	29	29	37	37
Num. arom. heavy atoms	6	12	12	18	18	24
Fraction Csp3	0	0	0	0	0	0
Number of rotatable bonds	2	2	6	5	8	7
Number of H-bond acceptors	3	4	7	8	10	11
Number of H-bond donors	2	2	2	5	2	5
TPSA (Å^2)	61.69	65.18	143.95	181.68	162.88	200.61
Consensus Log P	1.56	2.72	1.56	2.93	3.24	4.59
Solubility class	soluble	soluble	moderately soluble	moderately soluble	poorly soluble	poorly soluble
GI absorption	high	high	low	low	low	low
BBB permeant	yes	yes	no	no	no	no
P-gp substrate	no	yes	no	no	no	no
CYP 1A2 inhibitor	yes	no	no	no	no	no
CYP 2C19 inhibitor	no	no	no	no	no	no
CYP 2C9 inhibitor	no	no	no	yes	no	yes
Skin permeation log Kp (cm/s)	-6.07	-7.06	-7.01	-6.24	-5.96	-5.39
Lipinski (Pfizer) filter	yes; 0 violation	yes; 0 violation	yes; 0 violation	yes; 0 violation	no; 2 violation: MW>500; NorO>10	no; 2 violation: MW>500; NorO>10
Ghoser filter	yes	yes	yes	no; 1 violation: WLOGP>5.6	no; 3 violation: MW>480; WLOP>5.6; MR>130	no; 3 violation: MW>480; WLOP>5.6; MR>130
Veber (GSK filter)	yes	yes	no; 1 violation: TPSA>140	no; 1 violation: TPSA>140	no; 1 violation: TPSA>140	no; 1 violation: TPSA>140

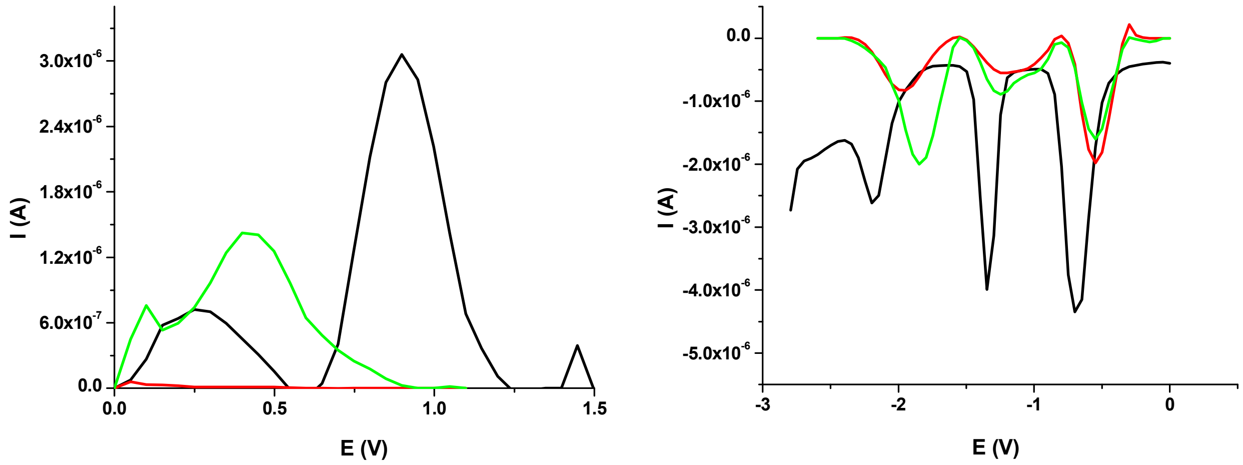

(**a**) (**b**)

Figure S1. DPV traces of M, B and T (0.001 M) in DMSO/Bu_4NBF_4 (0.1 M), potential domain. (**a**) (0.0 V - +1.5 V) anodic trace, (**b**) (0.0 V - +1.5 V) cathodic trace. DPV parameters were: SP=10 mV and MA=25 mV.

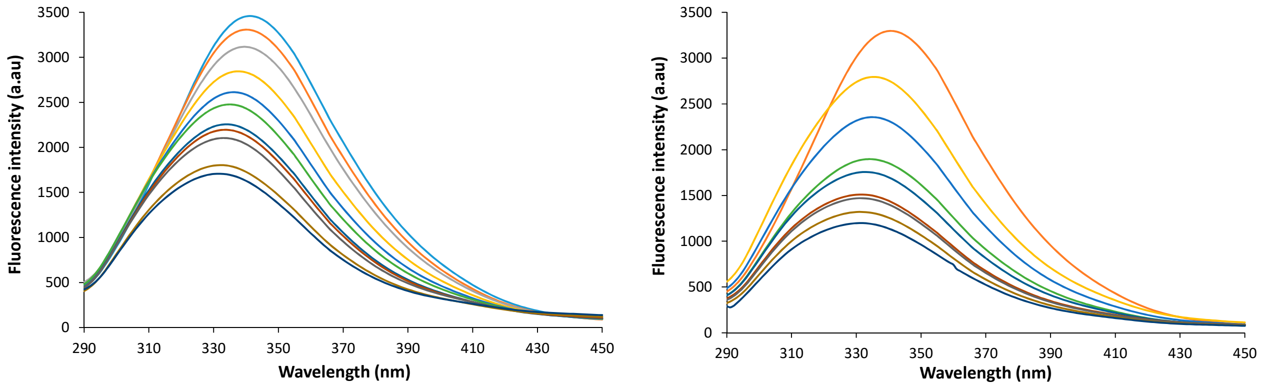

(**a**) (**b**)

Figure S2. Quenching of the BSA fluorescence (BSA 2 μM) in the absence and presence of various concentration of azo compounds: Mono_A (**a**) and Di_A (**b**) at 298 K. Concentrations of the quencher are in the range 0÷7 μM.

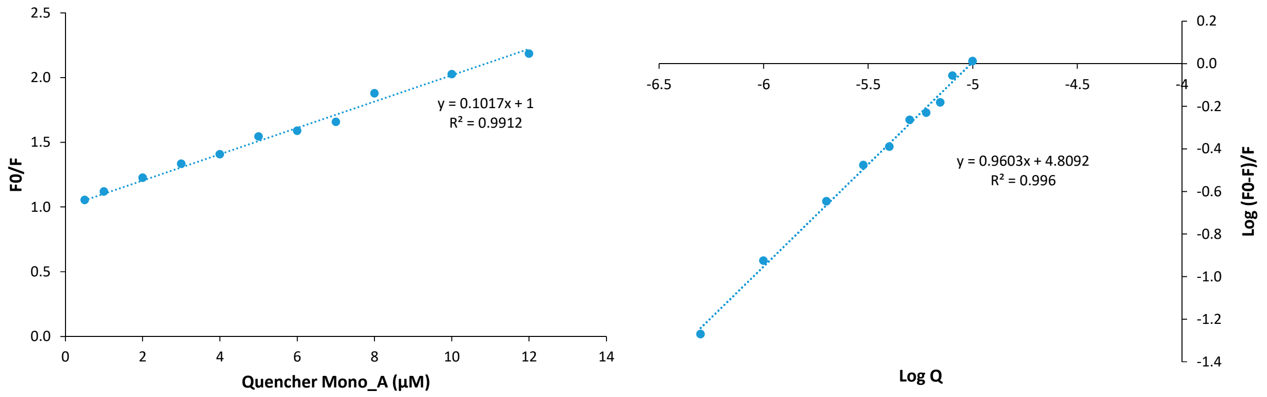

(**a**) (**b**)

Figure S3. Stern-Volmer plot (**a**) and double logarithmic plot (**b**) for fluorescence quenching in the BSA-azo derivative Mono_A solutions at 298 K.

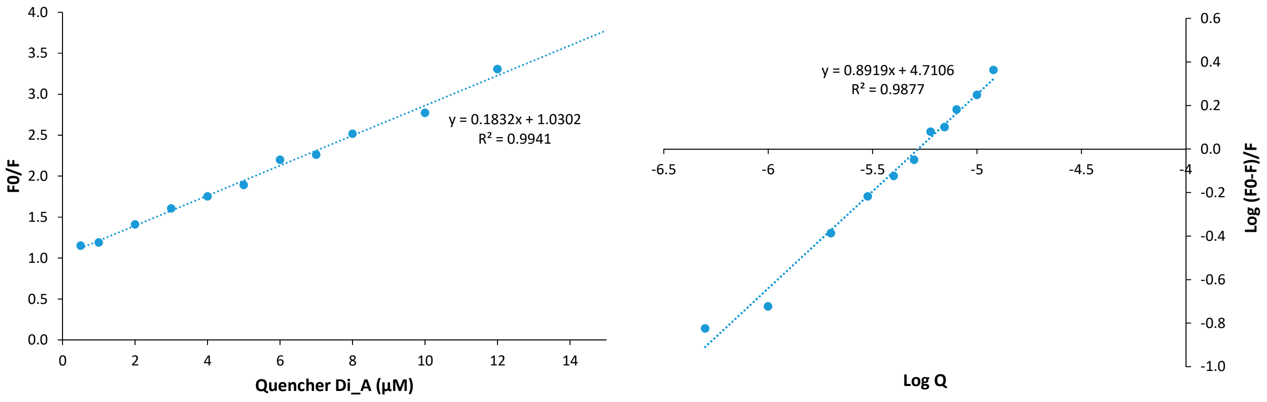

(**a**) (**b**)

Figure S4. Stern-Volmer plot (**a**) and double logarithmic plot (**b**) for fluorescence quenching in the BSA-azo derivative Di_A solutions at 298 K.

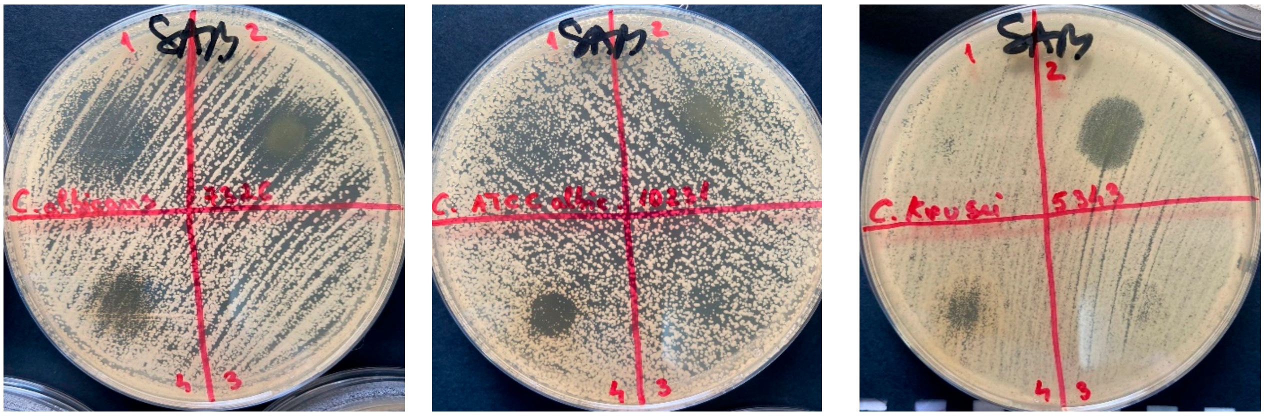

(**a**) (**b**) (**c**)

Figure S5. Inhibition zone aspect, after 24 hours of yeast strains incubation, in the contact with tested compounds. (a) *C. albicans* CL 7626, (b) *C. albicans* ATCC 10231 and (c) *C. krusei* CL 5343.
